# Proteomics Analysis Reveals Distinct Corona Composition on Magnetic Nanoparticles with Different Surface Coatings: Implications for Interactions with Primary Human Macrophages

**DOI:** 10.1371/journal.pone.0129008

**Published:** 2015-10-07

**Authors:** Carmen Vogt, Maria Pernemalm, Pekka Kohonen, Sophie Laurent, Kjell Hultenby, Marie Vahter, Janne Lehtiö, Muhammet S. Toprak, Bengt Fadeel

**Affiliations:** 1 Division of Molecular Toxicology, Institute of Environmental Medicine, Karolinska Institutet, Stockholm, Sweden; 2 Clinical Proteomics Mass Spectrometry, Department of Oncology-Pathology, Science for Life Laboratory, and Karolinska Institutet, Stockholm, Sweden; 3 NMR and Molecular Imaging Laboratory, Department of General, Organic and Biomedical Chemistry, University of Mons, Mons, Belgium; 4 Electron Microscopy Core Facility, Department of Laboratory Medicine, Karolinska Institutet, Stockholm, Sweden; 5 Division of Metals & Health, Institute of Environmental Medicine, Karolinska Institutet, Stockholm, Sweden; 6 Functional Materials Division, School of Information and Communication Technology, Royal Institute of Technology, Stockholm, Sweden; RMIT University, AUSTRALIA

## Abstract

Superparamagnetic iron oxide nanoparticles (SPIONs) have emerged as promising contrast agents for magnetic resonance imaging. The influence of different surface coatings on the biocompatibility of SPIONs has been addressed, but the potential impact of the so-called corona of adsorbed proteins on the surface of SPIONs on their biological behavior is less well studied. Here, we determined the composition of the plasma protein corona on silica-coated *versus* dextran-coated SPIONs using mass spectrometry-based proteomics approaches. Notably, gene ontology (GO) enrichment analysis and Kyoto Encyclopedia of Genes and Genomes (KEGG) pathway analysis revealed distinct protein corona compositions for the two different SPIONs. Relaxivity of silica-coated SPIONs was modulated by the presence of a protein corona. Moreover, the viability of primary human monocyte-derived macrophages was influenced by the protein corona on silica-coated, but not dextran-coated SPIONs, and the protein corona promoted cellular uptake of silica-coated SPIONs, but did not affect internalization of dextran-coated SPIONs.

## Introduction

Superparamagnetic iron oxide nanoparticles (SPIONs) have emerged as T2 contrast agents for magnetic resonance imaging (MRI) [1] and are also being considered as vehicles for drug delivery using MRI navigation [2]. Surface modification of SPIONs provides better chemical stability in biological fluids as well as increased circulation time in the blood [2]. The possibility to further modify the surface of the coated SPIONs enables increased biocompatibility and functionality of the nanoparticles [3]. We previously reported on the synthesis, magnetic properties and *in vitro* biocompatibility of monodispersed silica-coated, core-shell iron oxide nanoparticles (hereafter termed CSNPs) [4]. CSNPs were taken up more readily by macrophages when compared to commercially available dextran-coated SPIONs. The role of adsorbed proteins on the surface of the nanoparticles was not specifically assessed, but could play a role, as implied by several other studies [5]. Indeed, when nanomaterials confront physiological media, the adsorption of proteins or other biomolecules to the surface of the nanomaterials occurs, leading to the formation of a so-called bio-corona [5]. Protein corona formation will likely influence subsequent interactions of the particles with biological systems and consequently may affect their *in vivo* fate and distribution [6]. It has been suggested that the formation of a bio-corona on the surface of nanoparticles confers a new ‘biological identity’ to the nanoparticles [7,8]. This has obvious implications for nanomedicine and the administration of nanoparticles into the body as imaging and/or drug delivery agents. Formation of the bio-corona is governed by the primary size and surface properties such as surface charge, i.e., the ‘synthetic identity’ of the nanoparticles [9,10]. However, the nature of the protein corona is still, largely, unpredictable [11].

The aim of the present study was to determine how the surface of SPIONs affects the composition of the corona of human plasma proteins and the subsequent biological and magnetic behavior of the nanoparticles. To this end, comprehensive mass spectrometry-based proteomics assessment of the protein corona combined with bioinformatics data analysis was conducted for SPIONs of similar size with two different surface coatings (i.e., silica *versus* dextran). We also determined the magnetic relaxivity of the SPIONs with or without a plasma protein corona as well as the *in vitro* biocompatibility and cellular uptake of the nanoparticles using primary human macrophages. The present findings further our understanding of the role of the plasma protein corona for the behavior of magnetic nanoparticles intended for clinical applications such as MRI.

## Materials and Methods

### Nanomaterial synthesis and characterization

Dextran-coated SPIONs (hereafter named Nanomag-D-spio) were purchased from Micromod Partikeltechnologie GmbH Rostock-Warnemuende, Germany). The synthesis of silica-coated iron oxide core-shell nanoparticles (CSNPs) was performed as previously described [12] (and see S1 File for a detailed description). Detailed physico-chemical characterization, including inductively coupled plasma-optical emission spectrometry (ICP-OES), transmission electron microscopy (TEM), dynamic light scattering (DLS), and zeta potential measurements (with or without a pre-formed ‘hard’ corona of plasma proteins), was performed as described in S1 File.

### Magnetic resonance (MR) relaxometry measurements

Longitudinal (R_1_) and transverse (R_2_) relaxation rate measurements at 0.47 and 1.41T were obtained on Minispec Mq 20 and Mq 60 spin analyzers (Bruker, Karlsruhe, Germany) and nuclear magnetic relaxation dispersion (NMRD) profiles were recorded on a Spinmaster-FFC 2000 relaxometer (Stelar SRT, Mede, Italy). The measurements were performed on 300 μL of aqueous suspensions of the different nanoparticles with concentrations in the range of 2–7 mM/L Fe.

### Cellular studies and protein corona separation

**Fig 1:**
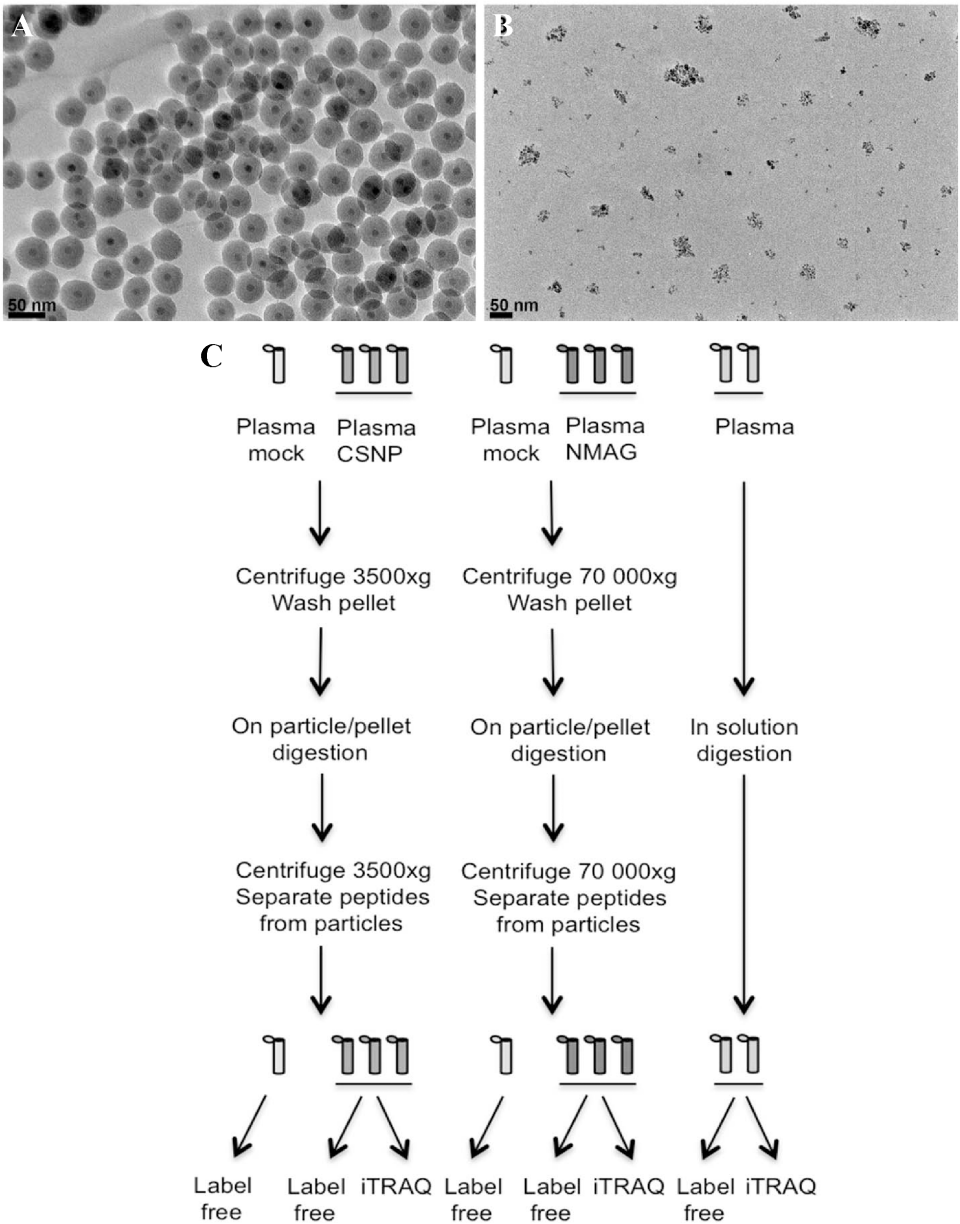
TEM of the silica-coated SPIONs (CSNP) (A) and dextran-coated SPIONs (nanomag-D-spio) (B). (scale bar = 50 nm). (C) Schematic overview of the protein corona analysis.

Human monocyte-derived macrophages (HMDMs) were isolated from buffy coats obtained from healthy blood donors (Karolinska University Hospital, Stockholm, Sweden) as described [13] (see S1 File for a detailed description). The nanoparticles were controlled for lipopolysaccharide (LPS) contamination prior to biological experiments by using the chromogenic LAL test method (Limulus Amebocyte Lysate endochrome, Charles River Endosafe, Charleston, SC). The LPS levels were always below 50 pg/mL. Cell viability was assessed by measurement of mitochondrial function using 3-(4,5-dimethyldiazol-2-yl)-2,5 diphenyl-tetrazolium bromide (MTT) (Sigma Aldrich), as described previously [4]. No FBS was added to the cell culture medium when experiments were conducted with nanoparticles with or without a plasma protein corona (the viability of HMDMs in the absence of FBS was verified in pilot experiments). TNF-α release was determined by ELISA (Mabtech, Nacka, Sweden) according to the manufacturer's instruction. Cellular uptake of nanoparticles with/without a protein corona was monitored by TEM. For quantification of cellular internalization of nanoparticles, cellular iron content was measured by inductively coupled plasma-mass spectrometry (ICP-MS), as described previously [14]. For protein corona studies, plasma from 14 healthy adult blood donors at the Karolinska University Hospital, Stockholm, Sweden was pooled, aliquoted and stored at −80°C until used. This pooled plasma was used throughout the study. For a detailed protocol for the nanoparticle incubation with plasma and subsequent steps to obtain the nanoparticle-protein corona complexes, refer to S1 File and see also Fig 1. Negative staining using phosphotungstic acid (PTA) with or without fixation with glutaraldehyde (GA), was employed for the visualization of the protein corona by TEM (see S1 File).

### Ethics statement related to human samples

As mentioned above, cells were isolated from buffy coats obtained from healthy adult blood donors at the Blood Transfusion Center, Karolinska University Hospital, Stockholm, Sweden. The donors are approved and covered by insurance according to the regulations at the University Hospital. These buffy coats contain white blood cells and are a waste product after the red blood cells have been utilized for blood transfusions. Similarly, the pooled plasma samples were obtained from anonymous blood donors. The identity of the blood donors is thus unknown to the scientists performing the experiments. In addition, the proteomics data generated in this study are stored without any personal identifiers and cannot be traced back to the individual blood donors. We previously sought the advice of the Ethical Committee for Human Studies in Stockholm in this matter, and a statement was issued that there are no objections to studies of nanomaterials on cells derived from human buffy coats, since the data cannot be traced back to the individual blood donors; thus, no specific ethical permit is required (see 2006/900-31/3, and decision 2006/3:8).

### Mass spectrometry and bioinformatics analyses

On-sample digestion of the nanoparticle-protein corona complexes and subsequent sample preparation is described in S1 File (and see Fig 1C for a schematic overview). Labeling of samples using iTRAQ (isobaric tags for relative and absolute quantification) 8-plex labels was performed according to the manufacturer’s protocol (Applied Biosystems, Foster City, CA). The mass spectrometry analysis was performed essentially as described previously [15] (see S1 File for a detailed description). The resulting data were searched by Sequest [16] under the Proteome Discoverer 1.3.0.339 software (Thermo Scientific, Waltham, MA) against the Swissprot database. The proteomics data generated in the present study have been deposited to the ProteomeXchange Consortium (http://proteomecentral.proteomexchange.org) via the PRIDE partner repository [17] with the dataset identifier PXD000766. For details on the statistical analysis of the data (see S1 and S2 Tables), the procedures used for estimation of relative protein abundances (S3 Table), and for the clustering analysis (S4 Table), see S1 File. The statistical comparisons included CSNPs *versus* plasma, nanomag-D-spio *versus* plasma, and CSNPs *versus* nanomag-D-spio. KEGG (Kyoto Encyclopedia of Gene and Genomes) Pathway and Gene Ontology (GO) enrichment analysis (S5 and S6 Tables) was performed as described in S1 File, using protein lists from the clustering analysis (S4 Table) and from the statistical analyses (S2 Table). Significant GO categories and KEGG pathways from the CSNP *versus* plasma comparison were visualized as stacked bar plots, using relative abundances of the proteins (S5–S7 Tables). The height of the bar for each protein corresponds to its relative abundance.

## Results

### Characterization of nanoparticles and nanoparticle-protein corona complexes

The characterization of dextran-coated nanoparticles (nanomag-D-spio) (obtained from a commercial source) and silica-coated nanoparticles (CSNPs) (synthesized as previously described [12]) included TEM, DLS and surface charge measurements as well as magnetic evaluation studies. The CSNPs had an average size of 44 nm (± 3.5 nm) as determined by TEM and the nanomag-D-spio displayed an average size of 5.7 nm (±2.1 nm), in agreement with the data supplied by the manufacturer (Fig 1A and 1B). It should be noted that the architecture of the latter nanoparticles is different insofar as these particles appear as clusters of approximately 50 nm. To determine whether plasma proteins influence the behavior of the two nanoparticles, we performed *in vitro* incubation of nanoparticles with human plasma, followed by multiple centrifugation and washing steps, in order to obtain nanoparticles with a ‘hard’ corona, as described in the Methods section. We then assessed whether the formation of a protein corona affects the hydrodynamic size of the nanoparticles. The hydrodynamic size was measured in cell culture medium, without FBS. The hydrodynamic diameter of the CSNPs with a protein corona increased significantly (3-fold) when compared to CSNPs, while the increase in hydrodynamic diameter for nanomag-D-spio was less pronounced (Table 1). These effects might be explained by the presence of an adsorbed layer of proteins on the nanoparticles, but could also be due to the formation of agglomerates of nanoparticles during the centrifugation of particle-plasma protein complexes.

**Table 1:** Size and surface charge of SPIONs with/without plasma corona.

Particles	DLS hydrodynamic (nm)^a^	Zeta potential (mV)^a^
**CSNP**	124.8 (±4.8)	-19.92 (±0.86)
**CSNP@protein corona**	391.7 (±11.7)	-11.24 (±0.39)
**Nanomag-D-spio**	81.8 (±1.2)	-4.87 (±0.69)
**Nanomag-D-spio@protein corona**	111.1 (±8.01)	-9.79 (±0.4)

DLS values are measured in RPMI 1640 cell culture medium (pH = 7.4) without FCS at 37°C.

^a^Measurements were performed on a colloidal suspension of particles, mean values ± SD, n = 3.

The surface charge (zeta potential) of the nanomag-D-spio and CSNPs with and without the protein corona was also measured. In the absence of a corona, CSNPs exhibited a surface charge of -19,92 (±0,86) mV and nanomag-D-spio -4,87 (±0,69) mV (Table 1). These values are close to the surface charge of the same particles measured in deionized (DI) water, as we reported previously [4]. The plasma protein corona increased the zeta potential of the CSNPs to -11,24 (±0,39) mV while for nanomag-D-spio the value decreased to -9,79 (±0,4) mV (Table 1). In other words, the protein corona served to equalize the surface charge of the two different SPIONs.

Next, we employed TEM in an attempt to visualize the protein corona. For the TEM evaluation of these nano-hybrids (inorganic phase plus a biological component), the conventional negative staining methodology for biological samples cannot be directly applied. However, the ‘hard’ protein corona is clearly visible on the surface of the CSNPs following staining with PTA, likely due to the differences in density between the iron oxide core, the silica shell and the heavy metal (tungsten) stained protein corona (S1 Fig). Using fixation with 1% GA for 20 min prior to staining with PTA, a non-shrunken contour of the protein corona could be visualized on the surface of the nanoparticles, similar to the shape of the non-stained protein corona. The protein corona on the nanomag-D-spio-protein corona hybrids appeared to have a more diffuse structure when compared to the distinct layer on the surface of the silica-coated nanoparticles (S1 Fig).

### Impact of the plasma protein corona on magnetic properties of the nanoparticles

A key question is whether the magnetic properties of SPIONs are affected by the plasma protein corona. To this end, relaxivity studies were performed on the CSNPs and nanomag-D-spio with or without a protein corona. The r_2_ values at 20 MHz and 60 MHz for CSNPs with a protein corona and nanomag-D-spio with a protein corona were bigger than the r_2_ values for CSNPs and nanomag-D-spio without a protein corona (Table 2). The r_1_ value followed a different pattern for the two types of particles after the addition of protein corona. While r_1_ values of CSNPs with a protein corona decreased compared with the value for CSNPs at 20 MHz and 60 MHz, the r_1_ values for nanomag-D-spio with a protein corona increased at both frequencies compared with nanomag-D-spio. Consequently, the r_2_/r_1_ values increased with 57% at 20 MHz and 63% at 60 MHz for CSNPs with a protein corona compared with CSNPs while for nanomag-D-spio alone *vs*. nanomag-D-spio with a corona the values are virtually unchanged (Table 2).

**Table 2:** Relaxivity properties of SPIONs with/without plasma corona

Particles	r_1_ (s^-1^ mM^-1^)^a^		r_2_ (s^-1^ mM^-1^)^a^		r_2_/ r_1_	
	20 MHz	60 MHz	20 MHz	60 MHz	20 MHz	60 MHz
**CSNP**	1.43 (±0.04)	0.74 (±0.02)	27.38 (±0.1)	32.22 (±0.1)	19.14	43.54
**CSNP@protein corona**	0.90 (±0.02)	0.47 ^b^	30.15 (±0.2)	32.44 ^b^	33.5	69.02
**Nanomag-D-spio**	17.86 (±0.1)	7.14 (±0.1)	65.57 (±0.2)	72.11 (±0.1)	3.67	10.09
**Nanomag-D-spio@protein corona**	21.87 (±0.2)	7.76 (±0.1)	83.64 (±0.1)	85.79 (±0.3)	3.82	11.05

Relaxivity values are measured at 20 MHz (0.47 T) and 60 MHz (1.41 T) in PBS (37°C).

^a^Results presented as mean values ± SD, n = 3.

^b^CSNP@protein corona particles are unstable over the measurements time at 60 MHz.

### Impact of the plasma protein corona on macrophage cell viability and cytokine release

**Fig 2:**
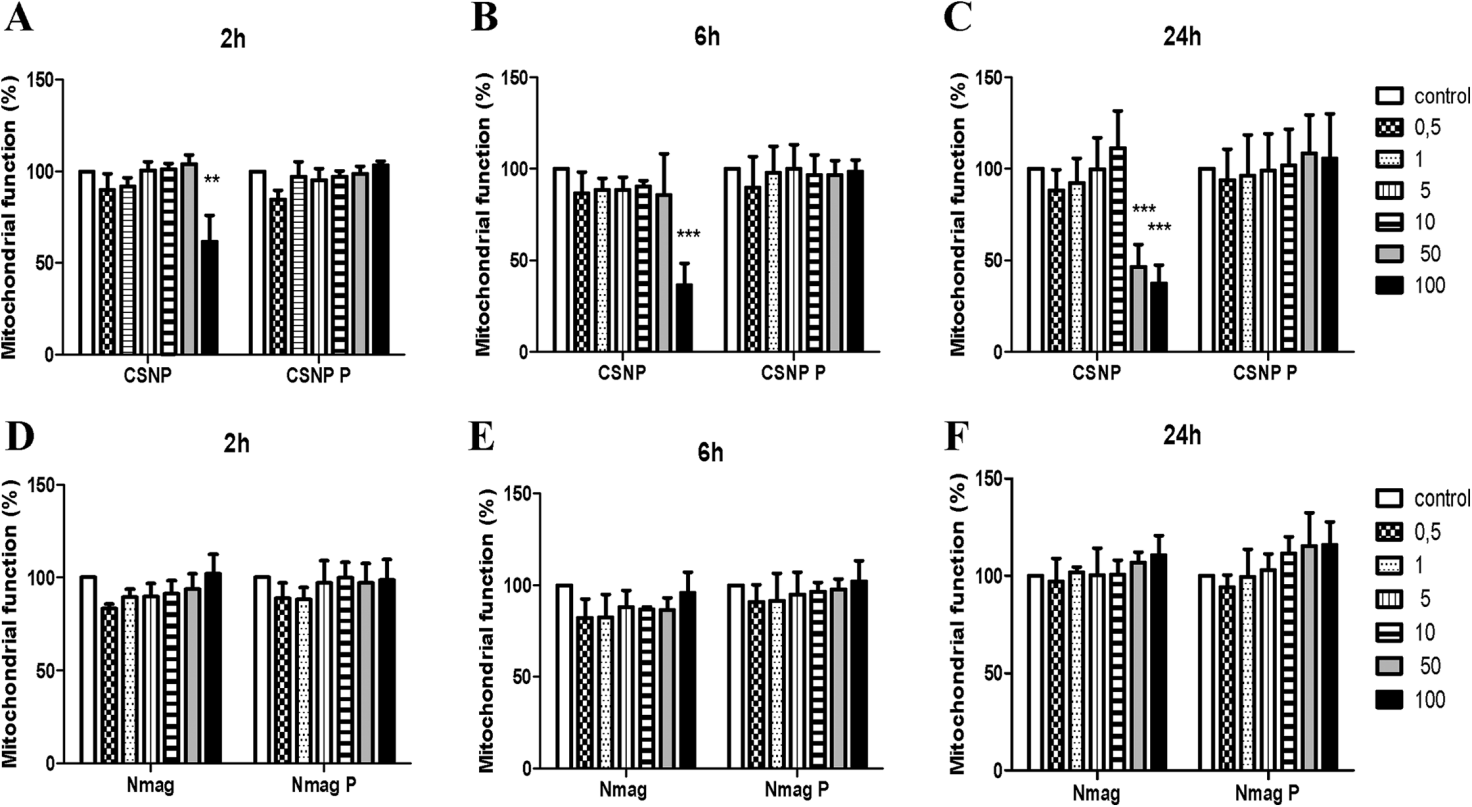
Biocompatibility assessment of SPIONs with or without a ‘hard’ corona of plasma proteins. Human monocyte-derived macrophages were exposed for 2 h (A, D), 6 h (B, E) or 24 h (C, F) to the indicated doses (μL/mL) of CSNP or CSNP + protein corona (A-C), or to nanomag-D-spio or nanomag-D-spio + protein corona (D-F). Macrophage viability was determined using the MTT assay. Cells were cultured in the absence of FBS to exclude any confounding effects of serum proteins. Results are presented as % mitochondrial function (mean values ± S.D.) from four independent experiments using cells isolated from healthy human donors. Statistical analysis was performed using Tukey post-hoc test following one way ANOVA (**p<0.01, ***p<0.001).

Nanoparticles that are intended for use as contrast agents in medical imaging are bound to come into contact with cells of the immune system and it is therefore of importance to study interactions of SPIONs with such cells [18], and to assess whether the protein corona may impact on toxicity or uptake of the nanoparticles. To this end, we performed biocompatibility studies using primary human monocyte-derived macrophages (HMDMs). Cell viability was assessed using the MTT assay, in the absence of FBS in the cell culture medium. As seen in Fig 2, cell viability decreased in a time- and concentration-dependent manner upon exposure to CSNPs; cell viability was thus decreased upon administration of nanoparticles at a concentration of 100 μg/ml, and at 24 h when exposed to 50 μg/ml of nanoparticles. Notably, biocompatibility was completely restored when HMDMs were incubated with CSNPs with a pre-formed ‘hard’ corona. In contrast, nanomag-D-spio were found to be non-cytotoxic for HMDMs, with or without a protein corona, at all doses and time-points tested. Others have shown that the protein corona (on gold nanoparticles) triggers the release of pro-inflammatory TNF-α in macrophage-like cells [19]. However, we could not observe any release of TNF-α after exposure of HMDMs to CSNPs or nanomag-D-spio, with or without a ‘hard’ corona of human plasma proteins, for up to 24 h (S2 Fig).

### Impact of the plasma protein corona on macrophage uptake of nanoparticles

**Fig 3:**
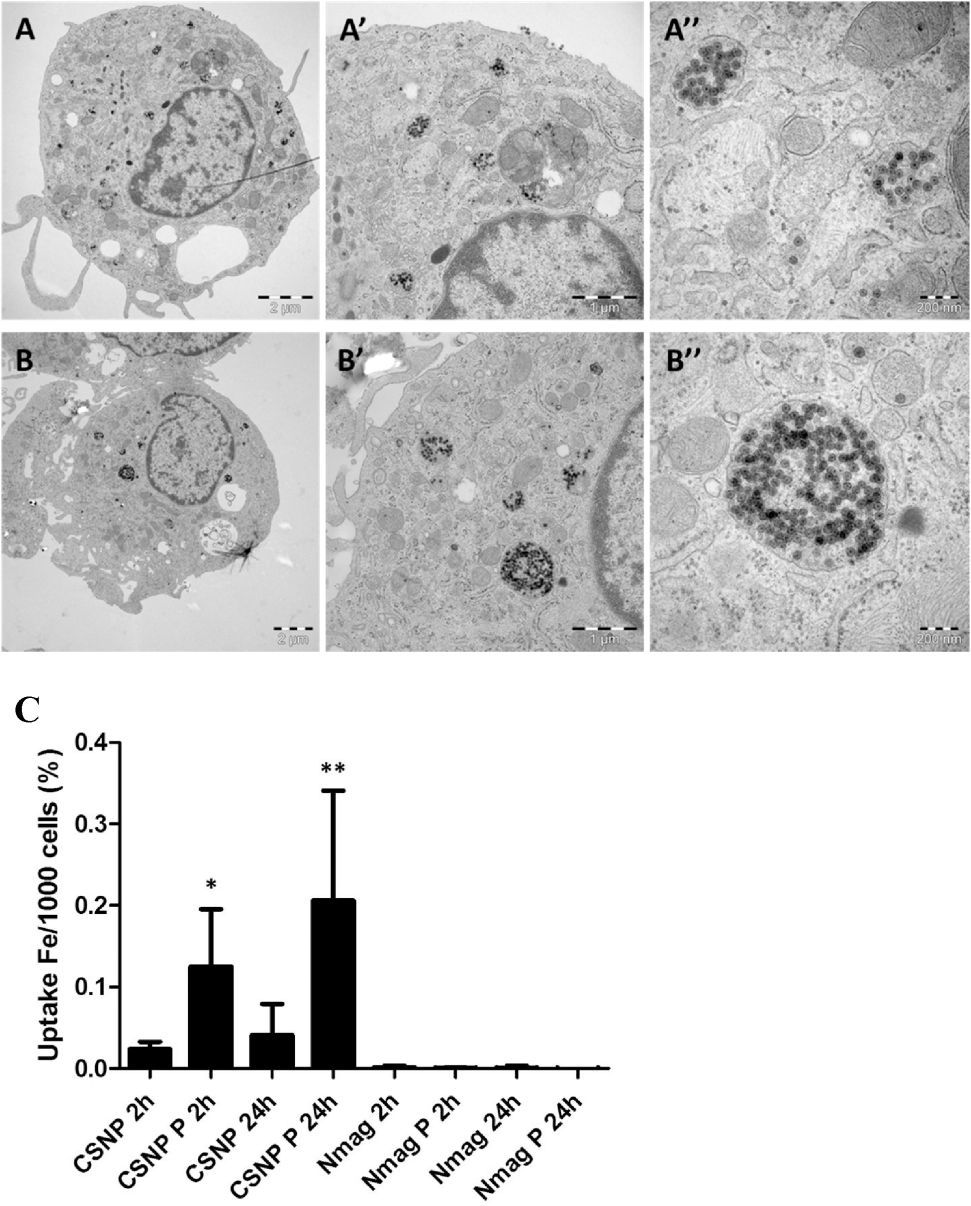
Macrophage internalization of SPIONs with or without a protein corona. TEM of HMDM exposed for 2 h to 50 μg/ml of CSNP (A-A”) or to CSNP + protein corona (B-B”). The arrows in (A, B) and (A’, B’) show some of the areas where the nanoparticles are located in the cells. In the higher magnification micrographs (A”, B”) the core-shell nanoparticles can be clearly seen. C. Uptake of CSNP, CSNP + protein corona, nanomag-D-spio, and nanomag-D-spio + protein corona was quantified by using ICP-MS to determine cellular iron content. HMDM were exposed to 50 μg/ml SPIONs for 2 h and 24 h. Results are presented as % of iron concentration per 1000 cells (mean values ± S.D.) adjusted to control, from three independent experiments, using cells from healthy donors. HMDM were maintained in cell culture medium without FBS. The difference in degree of cellular uptake between the two SPIONs in the absence or presence of a protein corona was evaluated using Tukey post-hoc test following one way ANOVA (*p<0.05,**p<0.01).

The internalization of CSNPs and nanomag-D-spio by HMDMs was determined using TEM and ICP-MS. These studies revealed marked differences in cellular uptake of the two nanoparticles. The nanoparticles were predominantly located in membrane-enclosed vesicles (presumed to be endosomes) and the number of particles within endosomes was greater in the case of CSNPs with a protein corona compared with CSNPs alone at 2 h of exposure (a time-point at which cellular toxicity can be excluded) (Fig 3A and 3B). Prolonged exposure (24 h) of cells to nanoparticles confirmed these differences (S3 Fig), demonstrating that the corona facilitates cellular uptake of CSNPs. Interestingly, higher magnification images of the internalized CSNPs and CSNP-protein corona complexes revealed that the morphology of the particles was very similar after internalization (Fig 3A and 3B); hence, while the silica shell and iron oxide core could be clearly seen in both cases, the protein corona on the surface of the CSNPs was not evident even though the staining protocol that was applied should also stain the organic (protein) layer on the surface of the CSNPs. However, recent studies have shown that while the ‘hard’ corona is retained on nanoparticles as they enter cells, it is subsequently degraded in lysosomes, thereby revealing the “naked” nanoparticles [20], an observation which could serve to explain the present findings. In contrast, macrophage uptake of nanomag-D-spio could not be seen at 2 h of incubation (data not shown) and some uptake was noted only after 24 h (S3 Fig). The presence of a protein corona did not appear to influence uptake of nanomag-D-spio. To corroborate these observations and in order to quantify the uptake of nanoparticles with and without a protein corona, ICP-MS was utilized, as previously described [4]. Macrophage uptake of silica-coated SPIONs (50 μg/ml) was significantly enhanced by the presence of a protein corona while uptake of dextran-coated SPIONs with or without a protein corona was very low (Fig 3C).

### Mass spectrometry-based analysis of the composition of the plasma protein corona

To determine whether the different surface coatings would influence the composition of the plasma protein corona, we performed LC-MS/MS mass spectrometry analysis on the protein corona extracted from SPIONs. A schematic overview of the analysis is shown in Fig 1C. Two different protein quantitation approaches were tested, a label-free quantitation based on spectral counting and a chemical labeling strategy using iTRAQ labels. Due to limited amount of material only 38 proteins were detected in the iTRAQ experiment compared with on average 128 proteins per sample from the label-free approach, and hence the downstream bioinformatics analyses were performed on the label-free data. The iTRAQ data was used to calculate the technical variability in the experiment. This calculation showed good reproducibility of the sample preparation (n = 3) and the average standard deviation of the relative protein amount of each of the proteins bound to the particles was 14% for CSNP and 16% for nanomag-D-spio (S8 Table), showing the robustness of the preparative method. In addition, our analysis of the corona retrieved from the label-free analysis showed a high reproducibility in terms of overlap of protein identification when comparing three replicates per SPION: 72% overlap of identified proteins for CSNPs and 73% for nanomag-D-spio (S4A and S4B Fig). To ensure that the enrichment of the protein corona was specifically due to binding of proteins to the particles and not due to unspecific enrichment during centrifugation, a mock plasma sample without particles was prepared in parallel with the CSNP and nanomag-D-spio protein corona (S4C Fig). As expected a higher number of proteins were identified from the nanomag-D-spio mock compared with the CSNP mock, in line with the higher centrifugation forces used when preparing the nanomag-D-spio protein corona (see Methods). This was further confirmed by the fact that all proteins that are identified from the lower centrifugation are also detected among the proteins from the higher centrifugation (S1 Table). However, the PSMs observed in the washed plasma tended to be much lower than the PSMs from corona samples, confirming the overall specificity of the nanoparticle associated protein binding. The protein corona retrieved from CSNPs had only albumin–the most abundant protein in plasma–and two other proteins in common with the mock, when taking into account proteins with at least 5 PSMs (S1 and S4 Tables).

### Comparative analysis of the corona on nanoparticles with different surface coatings

To investigate which plasma proteins were enriched on the two different particle types, relative to crude plasma, we performed statistical analysis of the peptide spectrum matches (PSMs) from the label free analysis. Based on this analysis we could detect 36 proteins, 19 of which had an estimated abundance above 0.5% using the top3 method, that were enriched on the CSNP particles relative to plasma and were not more abundant on the nanomag-D-spio particles according to our statistical analysis (Table 3, Table 4 and S2 Table, for lists see S9 Table). Using the same criteria, 64 proteins, of which 21 reached an abundance above 0.5%, were nanomag-D-spio enriched (Table 4). Estimation of the relative amounts of each of the proteins in the protein corona using the top3 method indicated that on average 60% of the total CSNP protein corona, excluding proteins that are more abundant on the nanomag-D-spio particles in a statistically significant manner, were enriched relative to the plasma proteome. Likewise, 54% of the nanomag-D-spio associated protein corona was distinct, indicating very different protein composition of the two nanoparticle coronas, both compared to each other and to crude plasma (S3 Table). The remaining corona associated proteins were not enriched relative to plasma, and therefore did not show specific association with the nanoparticles. The most abundant of the above CSNP specific proteins based on the top3 and statistical analysis results included fibrin precursor proteins fibrinogens alpha, beta and gamma as well as vitronectin and thrombospondin. Complement components C4A and C1QC as well as apolipoproteins E and B were also specifically associated with the CSNP particles, whereas the most abundant specifically bound proteins on the nanomag-D-spio particles included blood coagulation associated kininogen-1, microtubule associated serine/threonine kinase-like, platelet factor 4 and cytoplasmic actin 1 (Tables 3 and 4).

**Table 3:** Most abundant CSNP nanoparticle enriched proteins

UNIPROT	AAs	MW.kDa.	calc. pI	SYMBOL	GENENAME	log2 FC	CSNP	Nmag	Plasma	FDR q‐ value
**P02675**	491	55.9	8.27	FGB	fibrinogen beta	2.19	11.2%	0.4%	0.8%	2.7E‐02
**P02679**	437	49.5	6.09	FGG	fibrinogen gamma	1.72	9.8%	0.2%	0.7%	2.0E‐04
**P02671**	644	69.7	8.06	FGA	fibrinogen alpha	1.94	9.6%	0.2%	0.6%	4.3E‐04
**P04004**	478	54.3	5.8	VTN	vitronectin	1.68	5.8%	1.5%	0.5%	6.7E‐02
**P04196**	525	59.5	7.5	HRG	histidine‐rich glycoprotein	3.65	5.1%	1.7%	0.1%	5.8E‐04
**P07996**	1170	129.3	4.94	THBS1	thrombospondin 1	6.31	2.9%	1.3%	0.0%	4.0E‐06
**Q9HC10**	1230	140.2	6.24	OTOF	otoferlin	1.48	1.9%	0.0%	0.0%	1.7E‐01
**P00748**	615	67.7	7.74	F12	coagulation factor XII	5.54	1.7%	0.0%	0.0%	1.2E‐01
**P0C0L4**	1744	192.7	7.08	C4A/C4B	complement 4A / 4B	1.48	1.7%	0.5%	0.4%	7.6E‐01
**Q03591**	330	37.6	7.39	CFHR1	complement factor H‐ related 1	4.42	1.3%	0.0%	0.0%	1.1E‐01
**P02751**	2176	239.5	5.88	FN1	fibronectin 1	3.37	1.2%	0.2%	0.1%	6.5E‐01
**P00734**	622	70	5.9	F2	coagulation factor II	2.06	1.0%	0.0%	0.3%	7.9E‐03
**Q76FK4**	1041	117.4	6.92	NOL8	nucleolar protein 8	1.48	0.9%	0.0%	0.0%	2.0E‐01
**P00747**	810	90.5	7.24	PLG	plasminogen	1.56	0.7%	0.4%	0.3%	2.0E‐03
**P02649**	317	36.1	5.73	APOE	apolipoprotein E	3.77	0.7%	0.1%	0.1%	2.0E‐01
**P59665**	94	10.2	6.99	DEFA1	defensin, alpha 1/1B	4.19	0.6%	0.2%	0.0%	1.8E‐05
**P04114**	4563	515.3	7.05	APOB	apolipoprotein B	2.06	0.5%	0.0%	0.1%	1.0E‐01
**P02747**	245	25.8	8.41	C1QC	complement 1qC	3.49	0.5%	0.2%	0.0%	1.3E‐04

Uniprot = Uniprot accession, AAs = Number of Amino acids in the protein, MW.kDa. = molecular weight of the protein (calculated), calc.pI = protein isoelectric point (calculated), Symbol = Official gene symbol, GeneName = Official gene name, log2FC = log2 transformed fold‐change relative to plasma, CSNP% = relative abundance of the protein in the CSNP corona, Nmag% = NMag abundance, Plasma% = plasma abundance, FDR q‐value = false discovery rate corrected p‐value for the difference relative to plasma, CSNP = core shell nano particles; Nmag = nanomag‐D‐spio. Plasma = crude plasma (control).

**Table 4:** Most abundant nanomag-D-spio enriched proteins

UNIPROT	AAs	MW.kDa.	calc. pI	SYMBOL	GENENAME	log2 FC	CSNP	Nmag	Plasma	FDR q‐ value
**P01042**	427	47.9	6.65	KNG1	kininogen 1 microtubule associated ser/thr	2.6	2.3%	10.0%	0.3%	1.72E‐03
**Q96GX5**	840	92.8	6.13	MASTL	kinase‐like	1.0	1.0%	8.0%	0.0%	3.32E‐02
**P02776**	101	10.8	8.62	PF4	platelet factor 4	5.2	2.5%	6.7%	0.0%	7.00E‐06
**P60709**	375	41.7	5.48	ACTB	actin, beta	3.8	0.7%	3.6%	0.0%	2.47E‐04
**P08514**	953	103.2	5.67	ITGA2B	integrin, alpha 2b	5.3	0.5%	3.4%	0.0%	1.50E‐05
**P02775**	128	13.9	8.79	PPBP	pro‐platelet basic protein	0.0	1.3%	3.4%	0.3%	1.08E‐02
**P03952**	638	71.3	8.22	KLKB1	kallikrein B, plasma 1	2.8	0.5%	3.2%	0.0%	1.31E‐04
**P02788**	710	78.1	8.12	LTF	lactotransferrin	5.3	0.4%	2.3%	0.0%	4.00E‐06
**P13224**	206	21.7	9.31	GP1BB	glycoprotein Ib (platelet)	2.6	0.6%	1.7%	0.0%	3.40E‐05
**P05106**	780	86.1	5.27	ITGB3	integrin, beta 3	0.5	0.0%	1.6%	0.0%	4.90E‐04
**P21333**	2639	279.8	6.05	FLNA	filamin A, alpha	4.0	0.3%	1.0%	0.0%	7.00E‐06
**Q8WUI4**	614	66.1	6.57	HDAC7	histone deacetylase 7	‐0.6	0.0%	1.0%	0.0%	1.01E‐03
**P03950**	147	16.5	9.64	ANG	angiogenin	2.8	0.4%	0.8%	0.0%	2.73E‐02
**P18206**	1066	116.6	6.09	VCL	vinculin	1.9	0.1%	0.7%	0.0%	1.29E‐04
**Q9Y490**	2541	269.6	6.07	TLN1	talin 1	4.3	0.2%	0.6%	0.0%	7.00E‐06
**P68871**	147	16	7.28	HBB	hemoglobin, beta	‐0.6	0.0%	0.6%	0.0%	1.50E‐05
**P14770**	177	19	6.34	GP9	glycoprotein IX (platelet)	0.2	0.1%	0.5%	0.0%	3.37E‐03
**Q86UX7**	663	75.4	6.77	FE	fermitin family member 3	‐0.6	0.0%	0.5%	0.0%	1.80E‐05

Uniprot = Uniprot accession, AAs = Number of Amino acids in the protein, MW.kDa. = molecular weight of the protein (calculated), calc.pI = protein isoelectric point (calculated), Symbol = Official gene symbol, GeneName = Official gene name, log2FC = log2 transformed fold‐change relative to plasma, CSNP% = relative abundance of the protein in the CSNP corona, Nmag% = NMag abundance, Plasma% = plasma abundance, FDR q‐value = false discovery rate corrected p‐value for the difference relative to plasma, CSNP = core shell nano particles; Nmag = nanomag‐D‐spio. Plasma = crude plasma (control).

### Pathway and Gene Ontology (GO) analysis of the plasma protein corona

**Fig 4:**
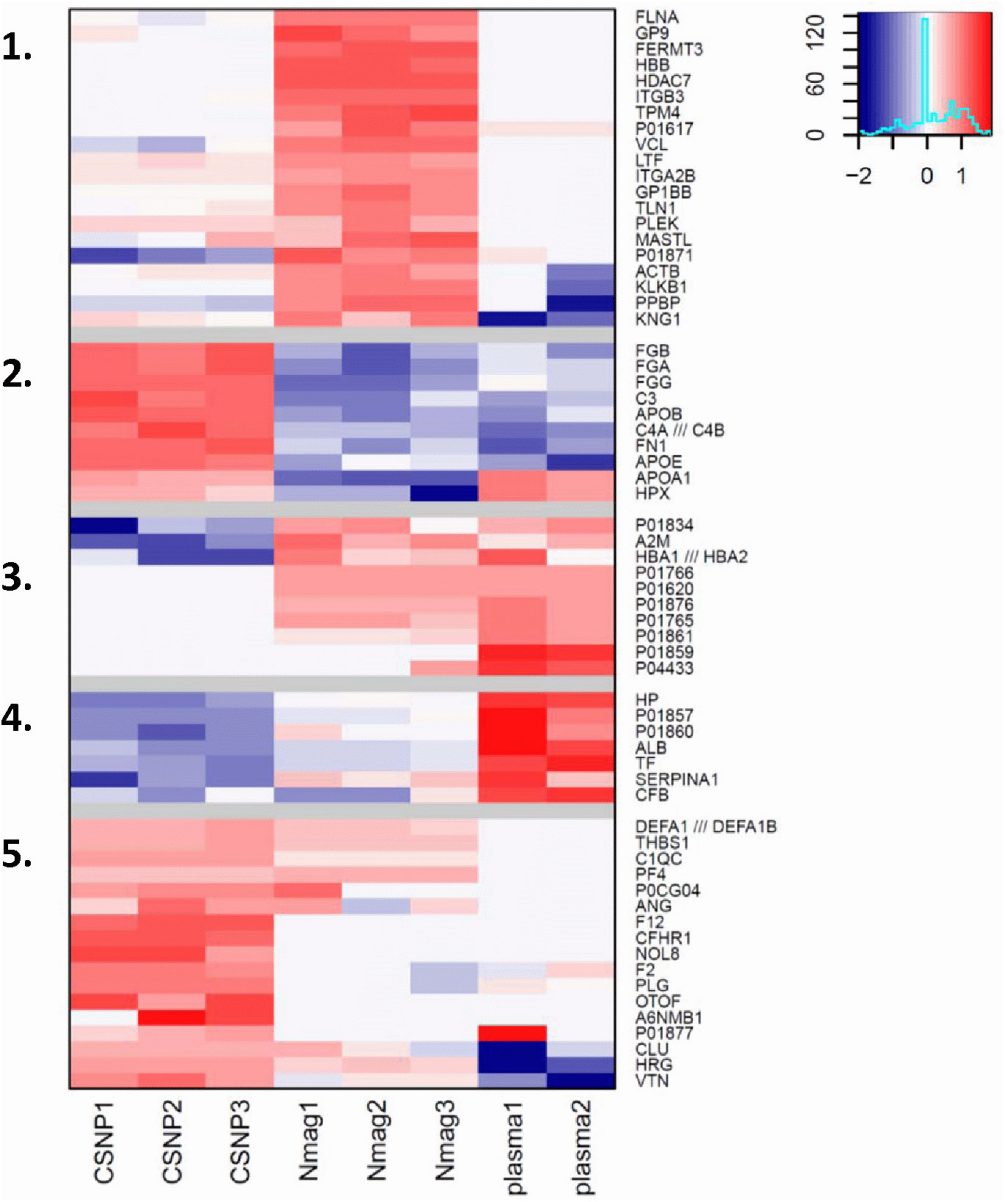
Distinct plasma protein corona composition on SPIONs with different surface coating. Cluster analysis of SPION-bound plasma proteins *versus* plasma proteins. Red color denotes counts higher than average when compared to other samples. Blue denotes counts lower than average (refer to legend top right corner). Clusters are numbered 1–5 (cf. S4 Table). Clusters 2 and 5 contain proteins that are enriched in the CSNP corona. Cluster 1 proteins are enriched in the nanomag-D-spio corona and clusters 3–4 contain plasma-enriched proteins. For gene ontology (GO) enrichment analysis of the corona-specific clusters, refer to Figs 5 and S5.

To detect functional subgroups or subclasses of proteins in the protein corona we performed clustering analysis and GO term enrichment based on the mass spectrometry data. Cluster analysis shows the data falling into 5 clusters (Fig 4). In cluster 1, proteins were almost exclusively found in the nanomag-D-spio protein corona while clusters 2 and 5 were enriched in the corona extracted from the surface of CSNPs. Clusters 3 and 4 contained proteins that are more abundant in crude plasma. Proteins with the strongest differential enrichment in the CSNP particle coronas, such as thrombospondin, histidine-rich glycoprotein and vitronectin, fell into cluster 5, whereas CSNP enriched proteins, such as fibrinogens, that are more abundant overall, but also present in lower amounts on other particles and in plasma could be found in cluster 2 (Tables 3 and 4).

**Fig 5:**
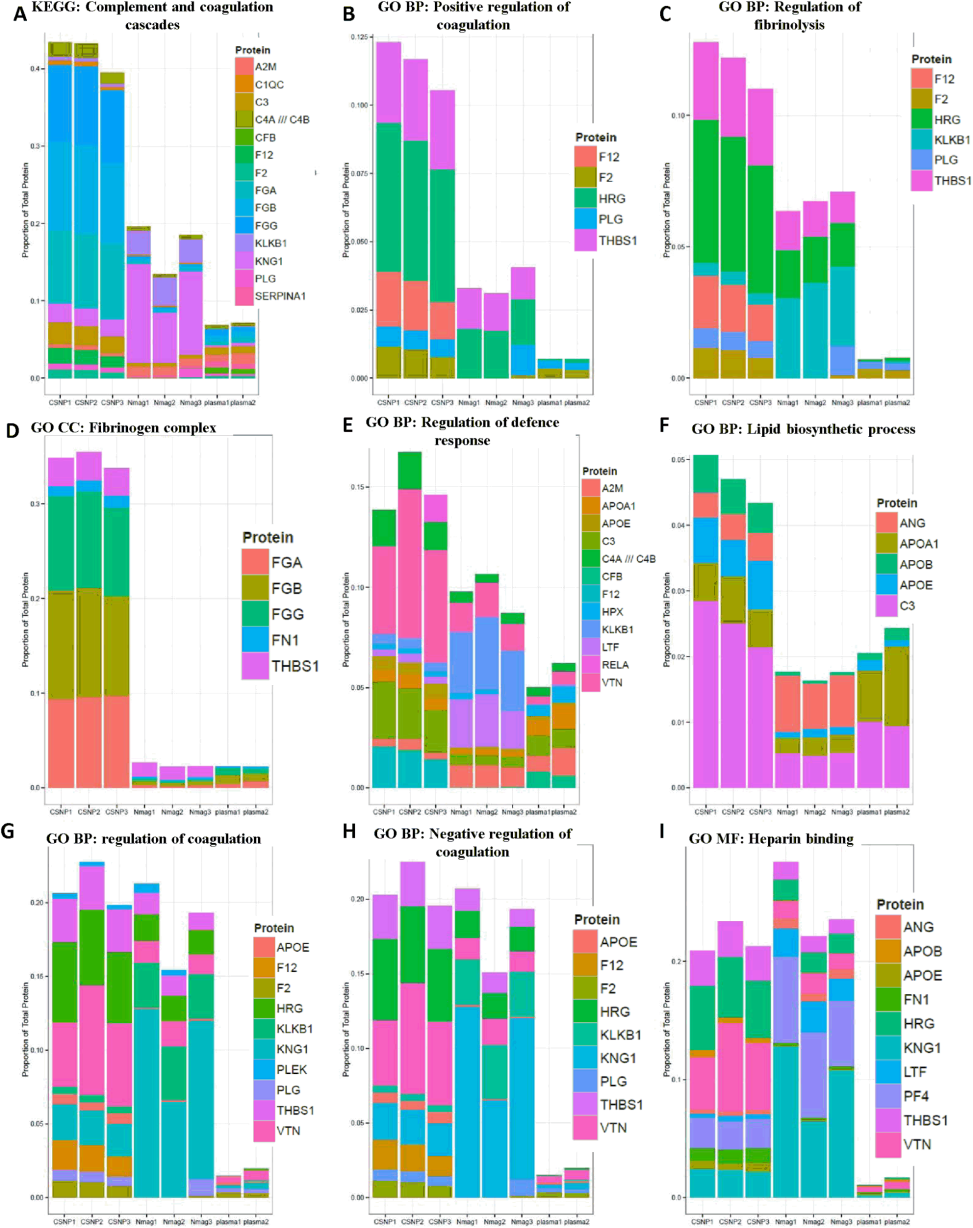
Plasma protein corona composition on CSNP. Classification of identified corona proteins according to differentially enriched Gene Ontology (GO) categories and KEGG pathways. The relative abundances of the proteins, as a percentage of total estimated protein abundance in the sample, are shown and each replicate is plotted separately. A significant enrichment of plasma proteins absorbed on the CSNP surface involved (A) KEGG Complement and coagulation cascades pathway, (B) GO Regulation of coagulation, (C) GO Negative regulation of coagulation, (D) GO Positive regulation of coagulation, (E) GO Regulation of fibrinolysis, (F) GO Fibrinogen complex, (G) GO Heparin binding, (H) GO Regulation of defense response, and (I) GO Lipid biosynthetic process. For further details, refer to S3, S5 and S6 Tables.

To further functionally classify the proteins identified in the study we performed GO enrichment analysis of the proteins in each cluster defined by the clustering analysis (Fig 4, S5–S7 Tables). Enriched GO terms included ‘fibrinogen complex’ and ‘lipid biosynthetic process’, that are associated with proteins belonging to cluster 2, as well as ‘regulation of coagulation’, ‘regulation of fibrinolysis’ and ‘heparin binding’, associated with proteins in cluster 5 (S5 Fig). In addition, we also carried out GO term enrichment analysis and KEGG enrichment analysis of the CSNP and nanomag-D-spio enriched proteins (S9 Table). Stacked bar plots were used to visualize the distribution of all the proteins annotated to a GO category enriched among proteins specifically associated with the CSNP corona (S5–S7 Tables), based both on the clustering analysis and on the statistical analysis (Fig 5). The estimated relative abundances for the 90 most abundant proteins were used for plotting (S4 Table). The KEGG pathway ‘complement and coagulation cascades’ related proteins as well as GO categories ‘positive regulation of coagulation’, ‘regulation of fibrinolysis’, ‘fibrinogen complex’, ‘regulation of defense complex’ and ‘lipid biosynthetic process’ were all enriched on the CSNP corona also by this measure (Fig 5), whereas ‘regulation of coagulation’, ‘negative regulation of coagulation’ and ‘heparin binding’ were only enriched according to the GO overlap analysis focused on statistically enriched protein identities and not by total abundance of all proteins belonging to the category (Fig 5).

## Discussion

The present study provides evidence that different surface coatings on SPIONs of similar overall size (approx. 50 nm) affects the composition of the corona of human plasma proteins. The protein corona, in turn, impacts on the magnetic properties of the silica-coated SPIONs and on their cellular uptake and on cytotoxicity, as evidenced in experiments using primary human macrophages, but does not appear to influence the behavior of the dextran-coated SPIONs. It should be noted that the current studies were performed using primary cells, not transformed cell lines; this may have a considerable impact on the outcome of the experiments and validity of the data. The apparent differences between the two SPIONs should be interpreted with caution, however, as the two particles display differences not only in surface coating, but also in structure (*i*.*e*., single core-shell nanoparticles with silica coating *versus* multiple iron core clusters with dextran coating). Nonetheless, the results obtained with the silica-coated CSNPs clearly showed that the plasma protein corona not only promotes cellular internalization, but also mitigates toxicity in the present model. Similarly, Dutta et al. [21] reported that the protein corona plays an important role in modulating cellular uptake and toxicity of SWCNTs and nano-sized amorphous silica in RAW264.7 macrophage-like cells. Tenzer et al. [22] reported that the formation of a corona of human plasma proteins promoted uptake of silica nanoparticles and positively charged polystyrene nanoparticles by endothelial cells, but did not affect uptake of negatively charged polystyrene nanoparticles to the same extent. During the preparation of this manuscript, Amiri et al. [23] reported that a protein corona composed of FBS affects the relaxivity of positively charged, dextran-coated SPIONs, but does not significantly affect SPIONs with neutral or negative charge. Thus, based on the latter work and on the data reported in the present study, it appears that the protein corona impacts on the magnetic behavior of SPIONs and that the surface coating (*i*.*e*., thickness/architecture, chemistry, surface charge) is an important determinant in this regard [24].

Walkey and Chan [7] compiled a list of identified plasma proteins and their relative abundances for more than 60 nanomaterials from two dozen published studies and were able to identify, across all studies, a total of 125 plasma proteins, representing the subset of plasma proteins that had been reported to adsorb to at least one nanomaterial. The physiological function of these proteins varied, but they were generally involved in lipid transport, blood coagulation, complement activation, pathogen recognition, or ion transport [7]. Indeed, in the present study, the protein corona extracted from CSNPs and compared to the plasma proteome and to the nanomag-D-spio protein corona was found to be enriched in proteins involved in almost all of these processes. Notably, complement, coagulation and immune defense related proteins are involved in opsonisation of microbes and their subsequent uptake by phagocytes, but also in systemic reactions such as thrombosis and anaphylaxis [25]. Furthermore, while apolipoproteins are thought to adsorb preferentially to hydrophobic nanoparticles, presumably due to hydrophobic interactions between the lipid part of the proteins and the nanoparticle surface [26], we found that apolipoproteins associated with both CSNPs and nanomag-D-spio, but that the lipid biosynthetic process was more prominently associated with silica coated SPIONs. For instance, apolipoprotein A1, a major component in high density lipoprotein complexes (HDL), was present in the protein corona extracted from both SPIONs. On the other hand, apolipoprotein E, a protein that is implicated in the transport of nanoparticles across the blood-brain barrier [27], was enriched on the surface of CSNPs. Furthermore, positive but not negative regulation of blood coagulation and regulation of fibrinolysis were also more prominently associated with the CSNPs.

One of the main findings in the present study is that the composition of the plasma protein corona on SPIONs depends on their surface coating. In line with this conclusion, recent studies focusing on three different carboxylic coating agents, citric acid, poly(acrylic acid), and oleic acid, also showed that differences in surface coating shape the protein corona composition of SPIONs [28]. Interestingly, in a very recent study, polyvinyl alcohol polymer (PVA)-coated SPIONs with negative and neutral surface charge adsorbed more serum proteins than dextran-coated SPIONs, which resulted in a longer blood circulation time in a rat model for the PVA-coated nanoparticles [29]. However, further studies are warranted in order to determine whether *specific* proteins may impact on the biodistribution of SPIONs or whether the adsorbed protein layer *per se*, irrespective of the identity of the individual proteins, serves to modify the behavior of the nanoparticles, for instance by affecting their agglomeration or surface charge. Ehrenberg et al. [30] provided evidence, using polystyrene nanoparticles and human umbilical vein endothelial cells, that cellular association is not dependent on the identity of adsorbed proteins and therefore unlikely to require specific binding to any particular cellular receptors. In line with these observations, Simberg et al. [31] concluded that candidate opsonins that had been identified in the corona of dextran-coated SPIONs did not play a significant role in the *in vivo* clearance of these particles. In fact, it has been stated that “there is no general rule that could be applied to every type of nanomaterial to predict the outcome [in terms of immune recognition]” [32]. However, the bioinformatics approach presented here provides a comprehensive methodology for obtaining particle corona-specific *signatures* comprised of functional categories, and has indicated specific enrichment of plasma proteins belonging to three categories, namely ‘blood coagulation regulation and fibrinogen complex’, ‘lipid biosynthesis’, and ‘immune defense’. Overrepresentation of these functional categories could thus constitute a signature or a *functional bio-identity* of nanoparticles that are readily recognized/internalized by macrophages, as demonstrated in the present study. Walkey et al. [33] characterized the serum protein corona composition for a library of 105 surface-modified gold nanoparticles and were able to develop a model that uses the corona composition to predict cell association more accurately than a model that uses parameters such as nanoparticle size, aggregation state, and surface charge. None of the proteins implicated in cellular uptake in the A549 cell line [33] are among the most differentially enriched proteins in the bio-corona in the present study. Nonetheless, while distinct models may be required for different classes of nanoparticles–and for different cell types–protein corona ‘fingerprinting’ could potentially be developed into a general strategy to predict the biological interaction of nanoparticles, as pointed out by the authors [33]. This may of particular relevance for our understanding of nano-interactions with the immune system [34].

## Conclusions

In conclusion, the present, comprehensive study has provided an illustration of the interplay between the synthetic and biological ‘identities’ of nanoparticles [8] and serves to underscore that the so-called corona of plasma proteins may influence both the magnetic and biological behavior of SPIONs, but also provides evidence that this is nanoparticle-dependent insofar as the corona was shown to promote cellular uptake of silica-coated, but not dextran-coated nanoparticles. Obviously, plasma proteins cannot be avoided when nanoparticles are administered intravenously in patients, and the grafting of PEG or other polymers onto nanoparticle surfaces may reduce, but will not completely prevent protein corona formation [35]. Therefore, the adsorption of proteins and other biomolecules needs to be taken into account in the design of any nanoparticle for clinical use, and the formation of a (specific) corona could also be exploited to confer novel advantageous properties to nanoparticles [11]. The present proteomics study has provided an inventory of human plasma proteins with affinity for silica- and dextran-coated SPIONs that may prove useful in further optimization of SPIONs for medical imaging.

## Supporting Information

S1 Fig Visualizing the protein corona. TEM micrographs of the ‘hard’ protein corona on CSNP and nanomag®-D-spio. CSNP + protein corona without staining (A), CSNP + protein corona with negative staining (B), CSNP + protein corona with fixation and negative staining (C), nanomag®-D-spio + protein corona without staining (D), nanomag®-D-spio + protein corona with negative staining (E), and nanomag®-D-spio + protein corona with fixation and negative staining (F).
(PPTX)

S2 Fig SPIONs ± corona do not trigger pro-inflammatory TNF-α secretion. Human monocyte-derived macrophages were exposed for 2 h (A, D), 6 h (B, E) or 24 h (C, F) to the indicated doses (μL/mL) of CSNP or CSNP + protein corona (A-C), or to nanomag®-D-spio or nanomag®-D-spio + protein corona (D-F). Cytokine release was assessed using ELISA. LPS was used as positive control. Results are presented as TNF-α release (pg/ml) (mean values ± S.D.) from three independent experiments using cells obtained from healthy blood donors. Statistical analysis was performed using Tukey post-hoc test following one way ANOVA (***p<0.001).
(PPTX)

S3 Fig Cellular uptake of SPIONs with or without a plasma protein corona. Primary human macrophages cultured without FBS were exposed for 24 h to 50 μg/ml of CSNP (A-A”), CSNP + protein corona (B-B”), nanomag®-D-spio (C-C”), and nanomag®-D-spio + protein corona (D-D”).
(PPTX)

S4 Fig Proteomics analysis of the plasma protein corona: good reproducibility. Good reproducibility in terms of overlap of protein identification was observed for the CSNP (A) and nanomag®-D-spio (B) corona. C. Venn diagram of CSNP and nanomag®-D-spio binding proteins compared to the corresponding mock plasma samples, i.e. plasma samples subjected to the same steps (see Fig 1C).
(PPTX)

S5 Fig Distinct plasma protein corona composition on the two different SPIONs. Gene ontology (GO) enrichment analysis of CSNP corona-specific, nanomag®-D-spio corona-specific and plasma-specific proteins based both on statistical analyses (see S2 Table) and on clustering (see Fig 4). Overrepresented GO categories related to each ‘signature’ (cf. S5 and S6 Tables) were hierarchically clustered. GO category branches are indicated as BP (Biological Process), MF (Molecular Function) and CC (Cellular Component). Cluster 1 proteins (nanomag®-D-spio enriched) are specifically enriched for GO ‘cell activation’ and GO ‘coagulation’, Cluster 2 (CSNP enriched) for GO ‘fibrinogen complex’ and GO ‘lipid biosynthetic process’, and Cluster 5 (CSNP) for GO ‘regulation of coagulation’, GO ‘heparin binding’ and GO ‘regulation of fibrinolysis’.
(PPTX)

S1 File Appendix A. Supplementary Materials and Methods.
(DOCX)

S1 Table Spectral counts (PSMs) of all proteins detected in the study. Uniprot = Uniprot accession (used for identification), Accession = Uniprot accession (original from proteomics analysis software), AAs = Number of Amino acids in the protein, MW.kDa. = molecular weight of the protein (calculated), calc.pI = protein isoelectric point (calculated), EntrezID = Entrez Gene identifier, Symbol = Gene Symbol, GeneName = Official gene name.; CSNP = core shell nano particles; Nmag = nanomag-D-spio. Plasma = crude plasma (control). Counts for some proteins from separate isoforms were combined, annotation information for each isoform was then indicated separately (with ///). Includes washed plasma controls for CSNP and nanomag-D-spio particles.
(XLSX)

S2 Table Statistical analysis of differential protein compositions identified in the respective nanoparticles’ coronas by quantitative label-free LC-MS. Data was filtered and counts-based analysis of 167 proteins carried out using R/Bioconductor limma/voom method, as described in material an methods. Comparisons included CSNPs versus plasma (csnp: csnp_vs_plasma), nanomag-D-spio versus plasma (nmag: nmag_vs_plasma), and CSNPs versus nanomag-D-spio (csnp.nmag: csnp_vs_nmag); Interpretation of the results: 1 = increased in comparison, 0 = not significant, -1 decreased in comparison. Threshold for statistical significance was set at q<0.05. Columns: A = log2 overall average of counts, Coef. = log2 fold-change for a comparison, t. = moderated t-statistic value for a comparison, p.value = p-value (limma/eBayes) for a comparison, p.value.adj = multiple testing adjusted p-value for a comparison, F = ANOVA F-statistic for the study, F.p.value = p.value of the F-statistic, F.p.value.adj = adjusted p-value of the F-statistic.
(XLSX)

S3 Table Estimated relative quantities for corona proteins identified by LC-MS for CSNP, nanomag-D-spio and for untreated plasma. For details of calculation, refer to Materials and Methods. CSNP = core shell nano particles; Nmag = nanomag-D-spio. Plasma = crude plasma (control).
(XLSX)

S4 Table Cluster analysis of nanoparticle coronas and plasma. Spectral counts (PSMs) were converted to Z-scores in a row-wise manner (columns starting with PSMz), as described in Materials and Methods. Clusters are numbered 1–5 (Cluster.pam). Data were plotted as a heatmap (Fig 4).
(XLSX)

S5 Table Gene Ontology (GO) category enrichment analysis results using the topGO R/Bioconductor package and the parentChild method. P-values were transformed (–log10(p-value). Columns: GO.ID = Gene Ontology identifier, Gobranch = GO branch (BP = biological process, MF = molecular function, CC = cellular component), Term = GO term name, totalSignif = total number of signatures where the p-value is below 0.01 (-log10(p-value)>2), minP = smallest p-value observed for a GO term. For descriptions of the signatures see Materials and Methods.
(XLSX)

S6 Table Detailed Gene Ontology (GO) category enrichment analysis results. Columns: ProteinList = signature used in the analysis (see Materials and Methods), GObranch = GO branch of the term (BP, MF or CC), GO.ID = GO identifier, Genes = Genes in the signature annotated to the GO term, Term = GO term name, Annotated = total number of genes annotated to the term, Significant = observed number of genes belonging to the signature annotated to the term, Expected = expected number of genes annotated to the term, Rank.in.classic = rank of the term using conventional GO term enrichment analysis, classic(-log10P) = -log10P of the p-value using conventional GO term enrichment analysis, p.c(-log10P) = -log10P of the p-value using the “parentChild” method for GO term enrichment analysis. For signature-associated genes, see S8 Table.
(XLSX)

S7 Table KEGG pathway enrichment analysis using the Webgestalt tool. Abbreviations: KEGG = Kyoto Encyclopedia of Genes and Genomes (http://www.genome.jp/kegg/). C = total number of proteins in the category/pathway, O = observed number of proteins in the category/pathway, E = expected number of proteins in the pathway. R = ratio between the expected and observed number of proteins in the category/pathway, rawP = p-value for enrichment calculated using the hyper geometric method, adjP = multiple testing corrected p-value (Benjamini-Hochberg method). Analysis parameters are recorded at the top of the table. For signature-associated genes, see S9 Table.
(XLSX)

S8 Table Number of identified proteins (at least one peptide with 99% confidence) and peptides (99% confidence) shown for each sample. Average standard deviation based on protein iTRAQ intensities is included for the preparation of the CSNP particles and nanomag-D-spio.
(XLSX)

S9 Table Gene lists used in the gene ontology and KEGG pathway overrepresentation analyses using Uniprot identifiers. (XLSX)
