# Supplementary material for: Proteomics Analysis Reveals Distinct Corona Composition on Magnetic Nanoparticles with Different Surface Coatings: Implications for Interactions with Primary Human Macrophages
Source: PLoS One. 2015 Oct 7;10(10):e0129008. doi: 10.1371/journal.pone.0129008 (PMC4596693; doi:10.1371/journal.pone.0129008)
Supplement: S1 Fig — TEM micrographs of the ‘hard’ protein corona on CSNP and nanomag®-D-spio. CSNP + protein corona without staining (A), CSNP + protein corona with negative staining (B), CSNP + protein corona with fixation and negative staining (C), nanomag®-D-spio + protein corona without staining (D), nanomag®-D-spio + protein corona with negative staining (E), and nanomag®-D-spio + protein corona with fixation and negative staining (F). (PPTX) [file pone.0129008.s001.pptx]

## Slide 1
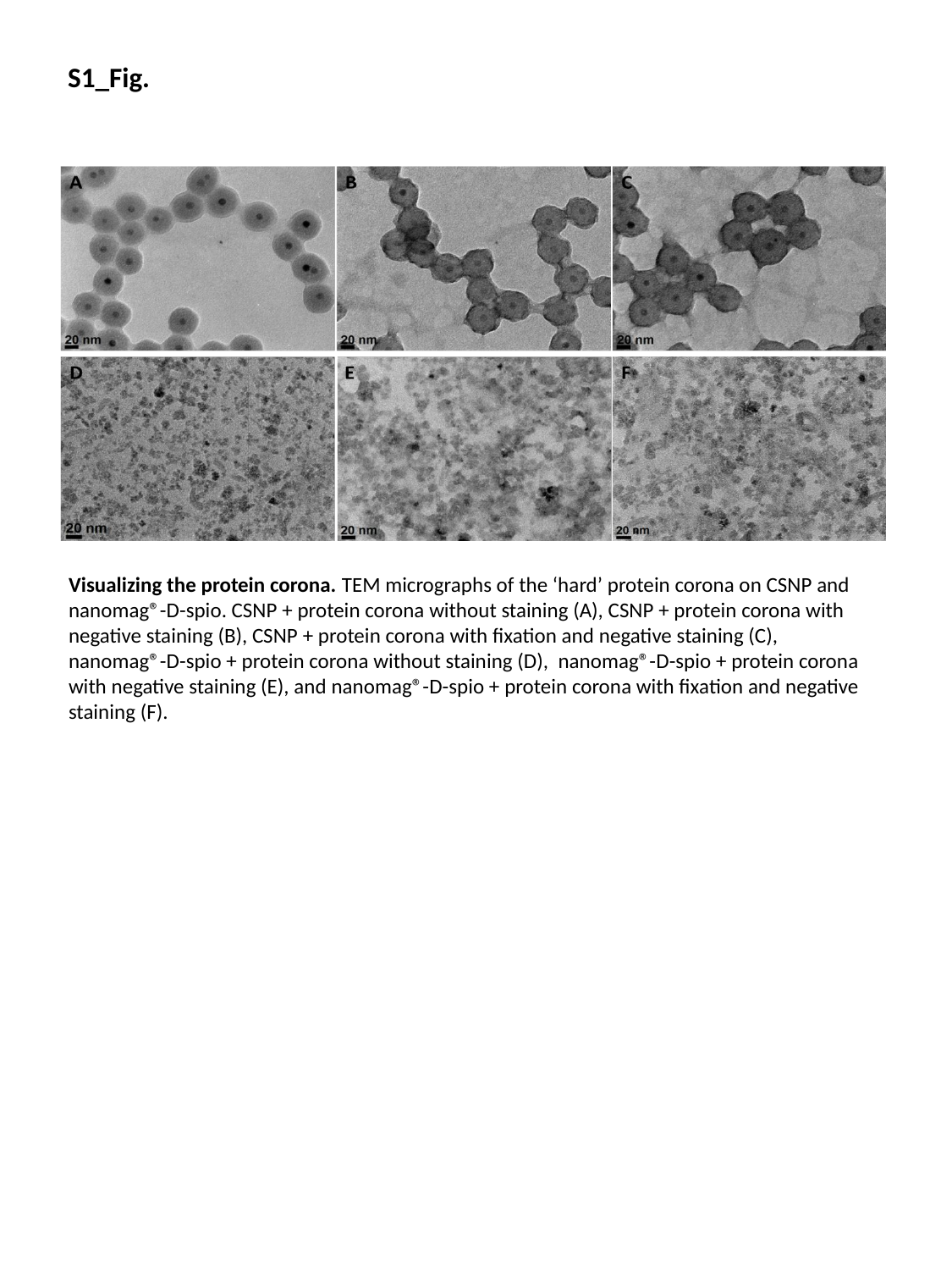

S1_Fig.
Visualizing the protein corona. TEM micrographs of the ‘hard’ protein corona on CSNP and nanomag®-D-spio. CSNP + protein corona without staining (A), CSNP + protein corona with negative staining (B), CSNP + protein corona with fixation and negative staining (C), nanomag®-D-spio + protein corona without staining (D), nanomag®-D-spio + protein corona with negative staining (E), and nanomag®-D-spio + protein corona with fixation and negative staining (F).
