# Supplementary material for: Proteomics Analysis Reveals Distinct Corona Composition on Magnetic Nanoparticles with Different Surface Coatings: Implications for Interactions with Primary Human Macrophages
Source: PLoS One. 2015 Oct 7;10(10):e0129008. doi: 10.1371/journal.pone.0129008 (PMC4596693; doi:10.1371/journal.pone.0129008)
Supplement: S2 Fig — Human monocyte-derived macrophages were exposed for 2 h (A, D), 6 h (B, E) or 24 h (C, F) to the indicated doses (μL/mL) of CSNP or CSNP + protein corona (A-C), or to nanomag®-D-spio or nanomag®-D-spio + protein corona (D-F). Cytokine release was assessed using ELISA. LPS was used as positive control. Results are presented as TNF-α release (pg/ml) (mean values ± S.D.) from three independent experiments using cells obtained from healthy blood donors. Statistical analysis was performed using Tukey post-hoc test following one way ANOVA (***p<0.001). (PPTX) [file pone.0129008.s002.pptx]

## Slide 1
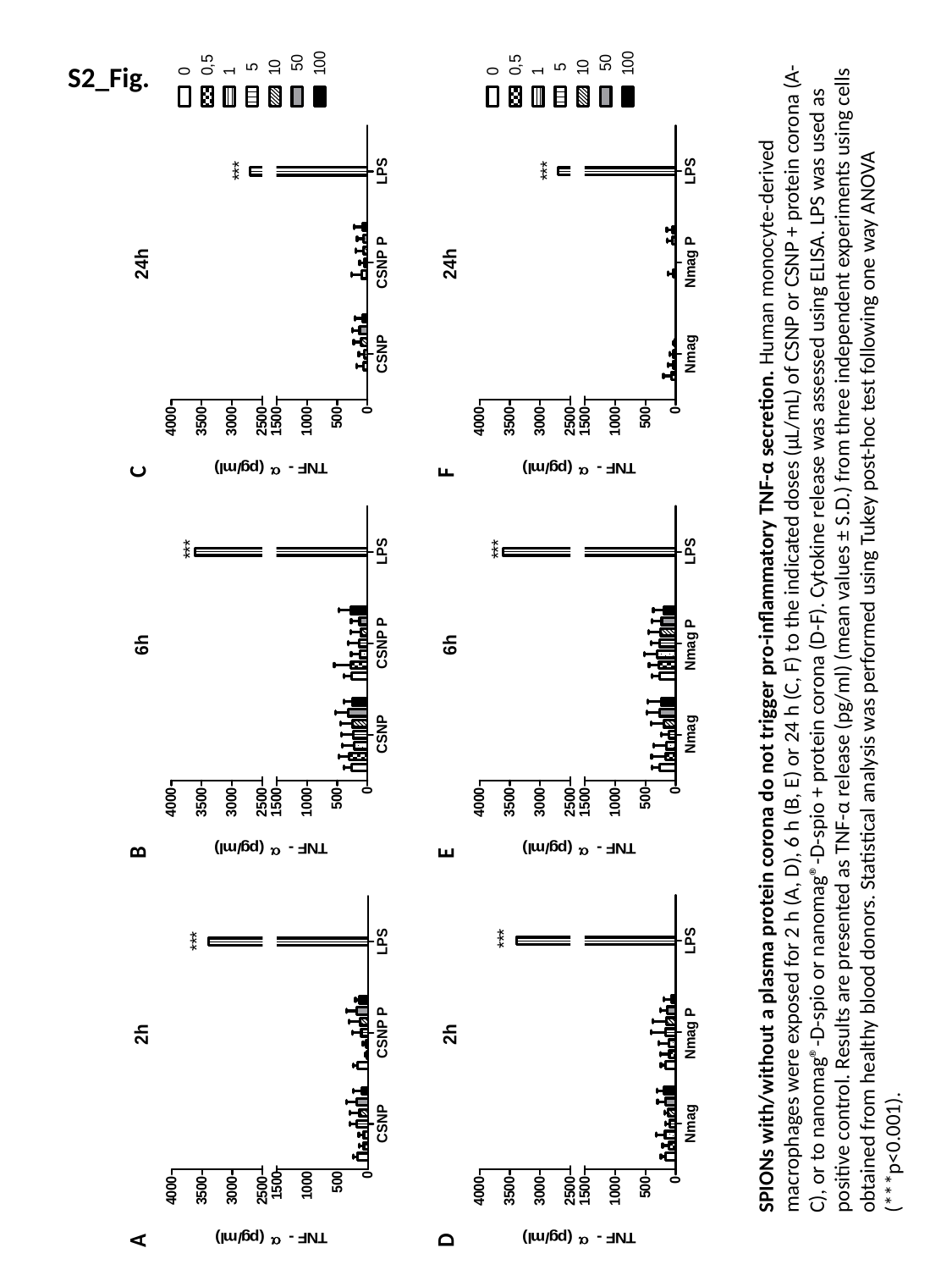

S2_Fig.
SPIONs with/without a plasma protein corona do not trigger pro-inflammatory TNF-α secretion. Human monocyte-derived macrophages were exposed for 2 h (A, D), 6 h (B, E) or 24 h (C, F) to the indicated doses (µL/mL) of CSNP or CSNP + protein corona (A-C), or to nanomag®-D-spio or nanomag®-D-spio + protein corona (D-F). Cytokine release was assessed using ELISA. LPS was used as positive control. Results are presented as TNF-α release (pg/ml) (mean values ± S.D.) from three independent experiments using cells obtained from healthy blood donors. Statistical analysis was performed using Tukey post-hoc test following one way ANOVA (***p<0.001).
