# Supplementary material for: Proteomics Analysis Reveals Distinct Corona Composition on Magnetic Nanoparticles with Different Surface Coatings: Implications for Interactions with Primary Human Macrophages
Source: PLoS One. 2015 Oct 7;10(10):e0129008. doi: 10.1371/journal.pone.0129008 (PMC4596693; doi:10.1371/journal.pone.0129008)
Supplement: S3 Fig — Primary human macrophages cultured without FBS were exposed for 24 h to 50 μg/ml of CSNP (A-A”), CSNP + protein corona (B-B”), nanomag®-D-spio (C-C”), and nanomag®-D-spio + protein corona (D-D”). (PPTX) [file pone.0129008.s003.pptx]

## Slide 1
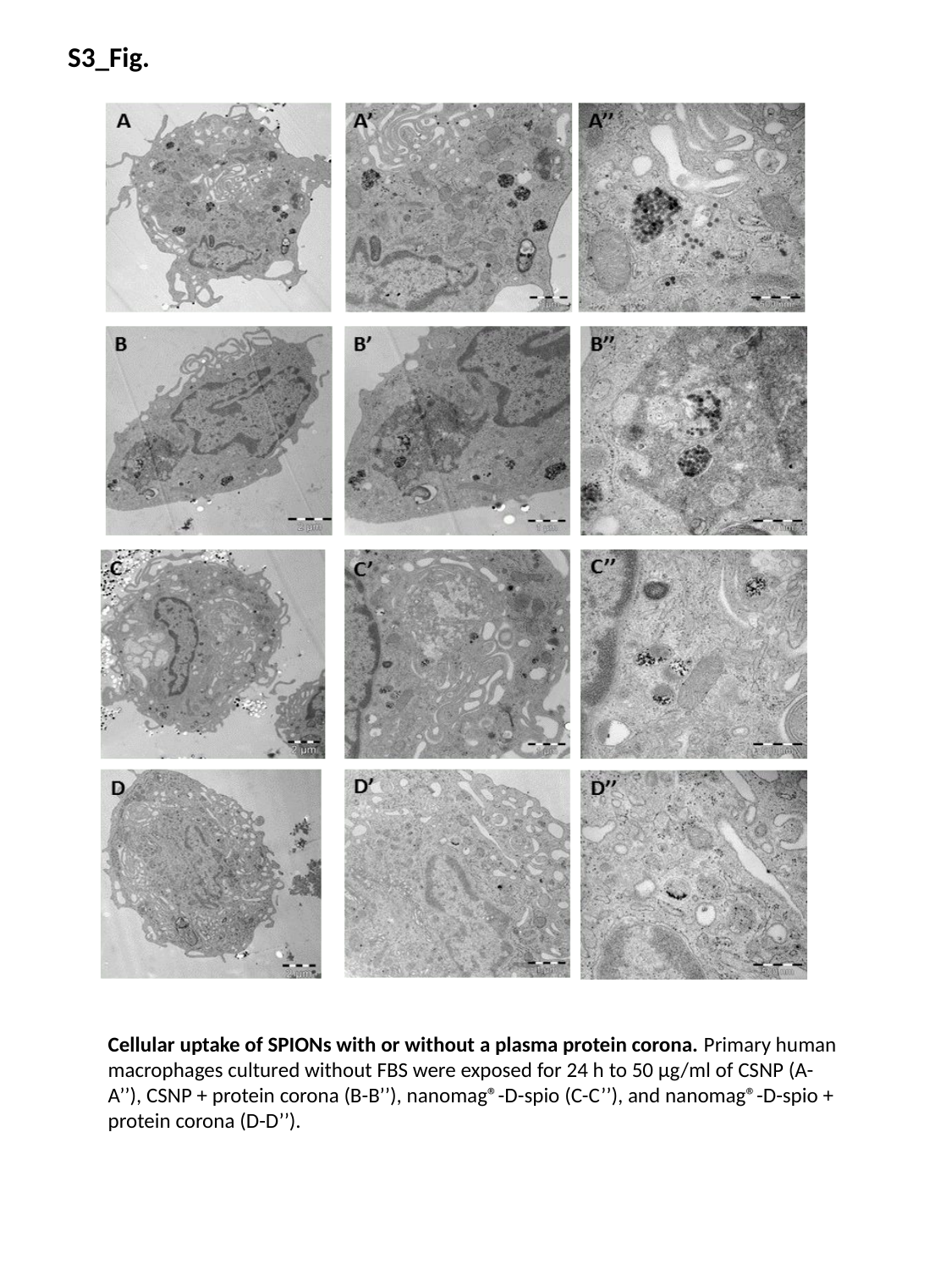

S3_Fig.
Cellular uptake of SPIONs with or without a plasma protein corona. Primary human macrophages cultured without FBS were exposed for 24 h to 50 µg/ml of CSNP (A-A’’), CSNP + protein corona (B-B’’), nanomag®-D-spio (C-C’’), and nanomag®-D-spio + protein corona (D-D’’).
