# Supplementary material for: Proteomics Analysis Reveals Distinct Corona Composition on Magnetic Nanoparticles with Different Surface Coatings: Implications for Interactions with Primary Human Macrophages
Source: PLoS One. 2015 Oct 7;10(10):e0129008. doi: 10.1371/journal.pone.0129008 (PMC4596693; doi:10.1371/journal.pone.0129008)
Supplement: S4 Fig — Good reproducibility in terms of overlap of protein identification was observed for the CSNP (A) and nanomag®-D-spio (B) corona. C. Venn diagram of CSNP and nanomag®-D-spio binding proteins compared to the corresponding mock plasma samples, i.e. plasma samples subjected to the same steps (see Fig 1C). (PPTX) [file pone.0129008.s004.pptx]

## Slide 1
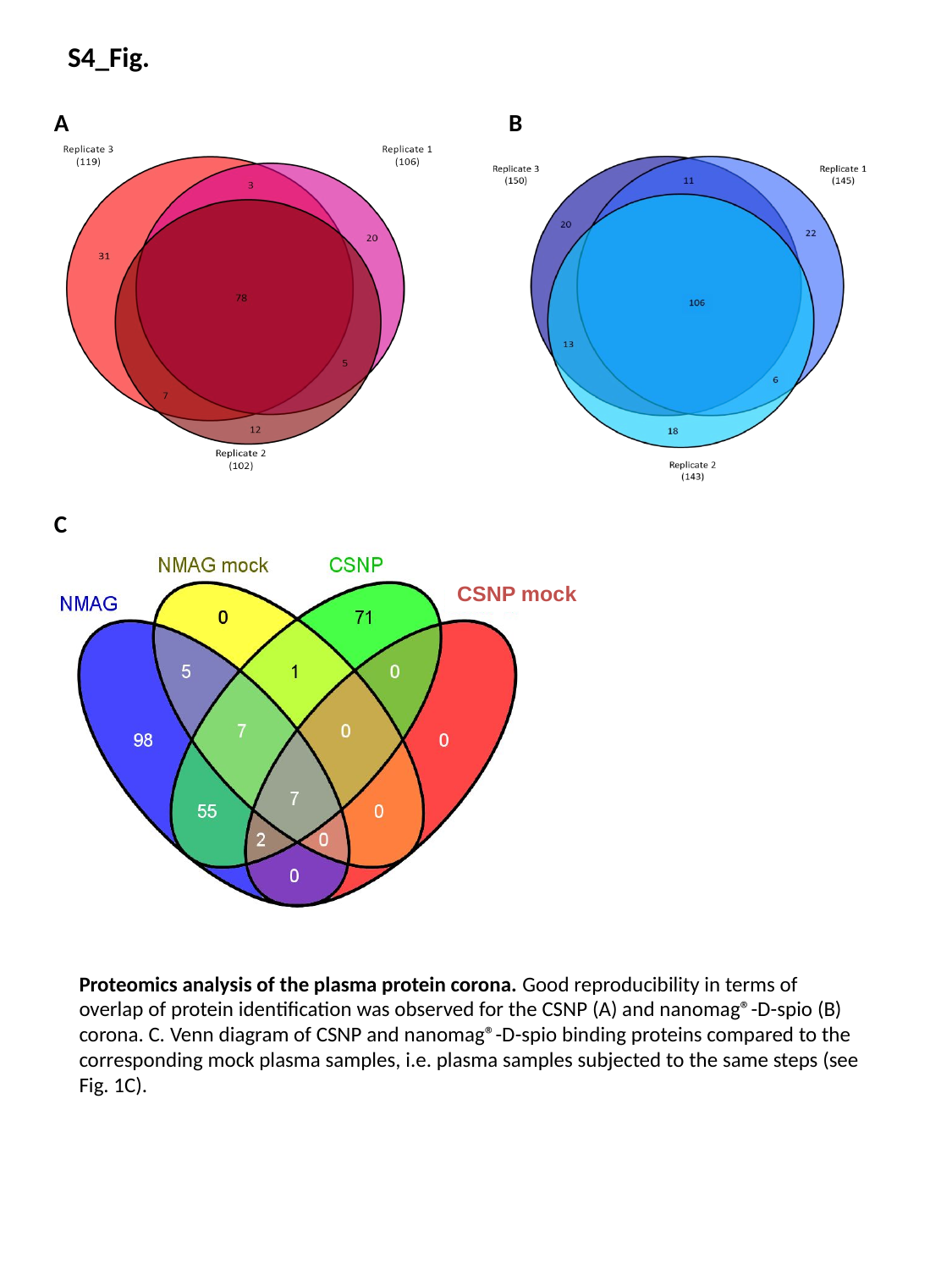

S4_Fig.
A
B
C
CSNP mock
Proteomics analysis of the plasma protein corona. Good reproducibility in terms of overlap of protein identification was observed for the CSNP (A) and nanomag®-D-spio (B) corona. C. Venn diagram of CSNP and nanomag®-D-spio binding proteins compared to the corresponding mock plasma samples, i.e. plasma samples subjected to the same steps (see Fig. 1C).
