# Supplementary material for: Proteomics Analysis Reveals Distinct Corona Composition on Magnetic Nanoparticles with Different Surface Coatings: Implications for Interactions with Primary Human Macrophages
Source: PLoS One. 2015 Oct 7;10(10):e0129008. doi: 10.1371/journal.pone.0129008 (PMC4596693; doi:10.1371/journal.pone.0129008)
Supplement: S5 Fig — Gene ontology (GO) enrichment analysis of CSNP corona-specific, nanomag®-D-spio corona-specific and plasma-specific proteins based both on statistical analyses (see S2 Table) and on clustering (see Fig 4). Overrepresented GO categories related to each ‘signature’ (cf. S5 and S6 Tables) were hierarchically clustered. GO category branches are indicated as BP (Biological Process), MF (Molecular Function) and CC (Cellular Component). Cluster 1 proteins (nanomag®-D-spio enriched) are specifically enriched for GO ‘cell activation’ and GO ‘coagulation’, Cluster 2 (CSNP enriched) for GO ‘fibrinogen complex’ and GO ‘lipid biosynthetic process’, and Cluster 5 (CSNP) for GO ‘regulation of coagulation’, GO ‘heparin binding’ and GO ‘regulation of fibrinolysis’. (PPTX) [file pone.0129008.s005.pptx]

## Slide 1
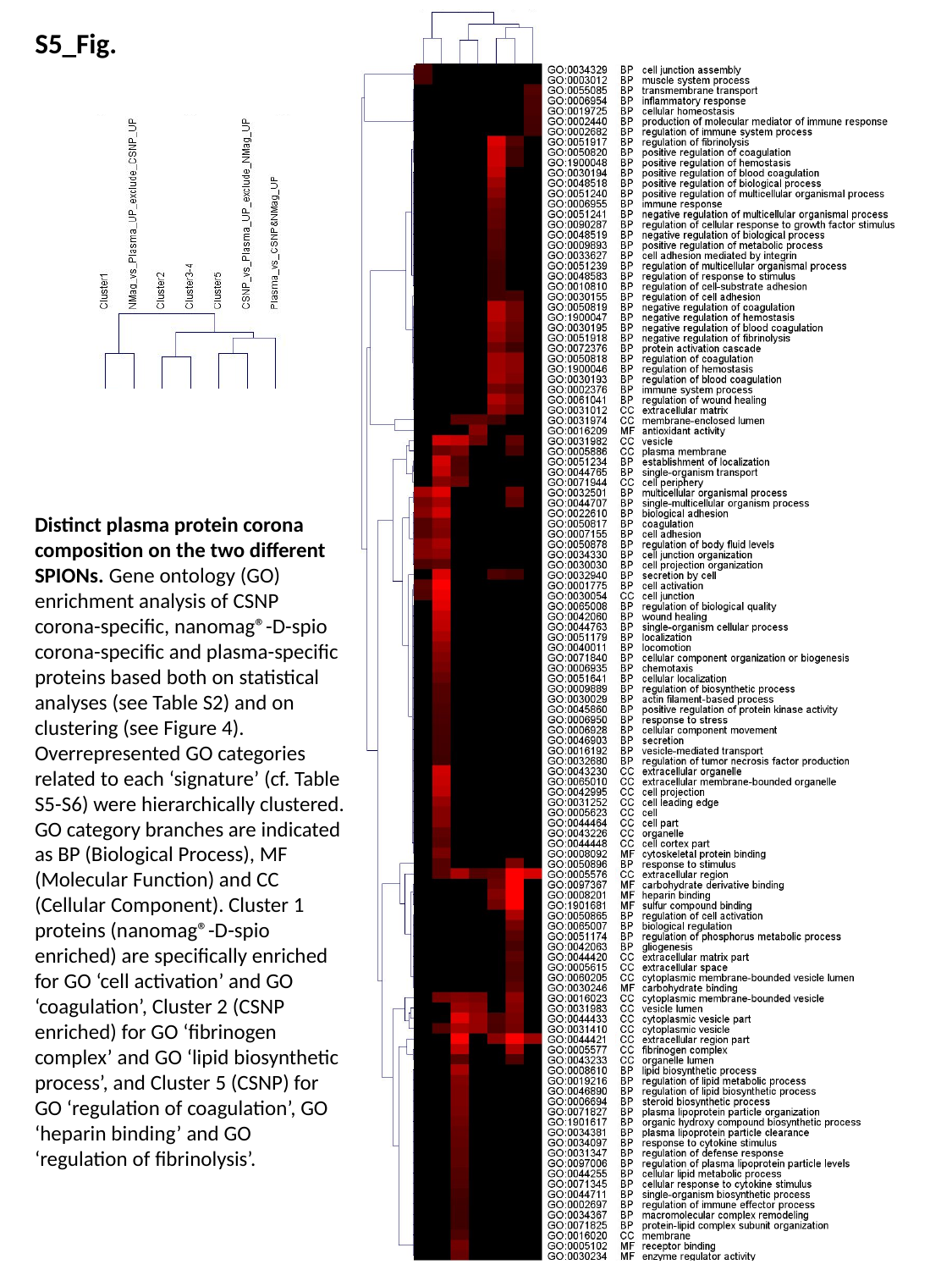

S5_Fig.
Distinct plasma protein corona composition on the two different SPIONs. Gene ontology (GO) enrichment analysis of CSNP corona-specific, nanomag®-D-spio corona-specific and plasma-specific proteins based both on statistical analyses (see Table S2) and on clustering (see Figure 4). Overrepresented GO categories related to each ‘signature’ (cf. Table S5-S6) were hierarchically clustered. GO category branches are indicated as BP (Biological Process), MF (Molecular Function) and CC (Cellular Component). Cluster 1 proteins (nanomag®-D-spio enriched) are specifically enriched for GO ‘cell activation’ and GO ‘coagulation’, Cluster 2 (CSNP enriched) for GO ‘fibrinogen complex’ and GO ‘lipid biosynthetic process’, and Cluster 5 (CSNP) for GO ‘regulation of coagulation’, GO ‘heparin binding’ and GO ‘regulation of fibrinolysis’.
