# Supplementary material for: Proteomics Analysis Reveals Distinct Corona Composition on Magnetic Nanoparticles with Different Surface Coatings: Implications for Interactions with Primary Human Macrophages
Source: PLoS One. 2015 Oct 7;10(10):e0129008. doi: 10.1371/journal.pone.0129008 (PMC4596693; doi:10.1371/journal.pone.0129008)
Supplement: S1 File — Supplementary Materials and Methods. (DOCX) [file pone.0129008.s006.docx]

**Plasma protein corona formed on silica-coated iron oxide nanoparticle has a distinct composition and influences the interactions with primary human macrophages and magnetic behavior**

Carmen Vogt^1,#a^, Maria Pernemalm^2^, Pekka Kohonen^1^, Sophie Laurent^3^, Kjell Hultenby^4^, Marie Vahter^5^, Janne Lehtiö^2^, Muhammet Toprak^6^, Bengt Fadeel^1,*^

**S1 File. Appendix A**

**Materials**

Dextran-coated SPIONs (hereafter named Nanomag®-D-spio) were purchased from Micromod Partikeltechnologie GmbH Rostock-Warnemuende, Germany). Iron chloride, FeCl_3_, and sodium oleate were purchased from Riedel-de Haën, Triton-X100 (analytical grade), cyclohexane (99.5%) and hexanol (98%), dioctyl ether (99%) were from Sigma Aldrich (St. Louis, MO) and oleic acid, tetraethyl orthosilicate (TEOS) (99.5%) and NH4OH (28%) from Fluka (Sigma Aldrich). Ethanol was of 99.9% purity and the water was MilliQ grade with a resistivity of 18 MΩ.

**Synthesis of silica-coated iron oxide nanoparticles**

The iron oxide nanoparticles were synthesized using a high temperature decomposition method in organic solvent [1]. The obtained oleic acid capped particles were separated by several cycles of precipitation with ethanol and dispersion in cyclohexane. The core particles were subsequently coated with a silica shell using an inverse microemulsion method (Triton-X100/hexanol/water/cyclohexane system) as previously described [2]. The resulting core-shell nanoparticles (CSNPs) with a size of 24.3 nm (±2.1 nm) were obtained after several cycles of cooling in liquid N_2_, centrifuging and dispersion in ethanol. To obtain particles with an average diameter 44 nm, the Stöber method was used [3]. In a typical experiment water and ethanol in a ratio of 2:3 were mixed with silica coated iron oxide particles with an overall diameter of 24.3 nm (±2.1 nm) obtained as described above. The pH of the reaction was adjusted to 8–9 with NH_4_OH. The particles were washed by several ultra-centrifugations at 2°C and re-dispersed in water.

**Physico-chemical characterization of nanoparticles**

**Inductively coupled plasma-optical emission spectrometry (ICP-OES).** A known volume of nanoparticle sample (CSNPs or nanomag®-D-spio, with or without a ‘hard’ protein corona; see corona section below) was dispersed in water and the concentration of Fe and Si was measured in triplicate using an ICP–Optical Emission Spectrometer, iCAP 6000 series (Thermo Scientific, Cambridge, UK). Fe and Si Accu Trace™ Reference Standard solutions for ICP (Analytical Standards AB, Mölnlycke, Sweden) were used. **Transmission electron microscopy (TEM).** A drop of nanoparticle suspension was air dried onto a carbon film coated TEM grid and analysis of nanoparticle size and morphology was performed using a JEM-2100 (JEOL Ltd., Tokyo, Japan) at 200 kV acceleration. The mean diameter and S.D. was calculated after measuring at least 300 nanoparticles per sample in random visual fields. **Dynamic light scattering (DLS).** The hydrodynamic diameter of the nanoparticles with/without a protein corona was measured using the Beckman-Coulter DelsaNanoC system (Beckman Coulter Inc., London, UK). The measurements were performed at 37°C in RPMI-1640 cell medium without fetal bovine serum (FBS) (pH 7.4) on a Zetasizer Nanoseries Zen 3600 (Malvern, UK). **Zeta potential.** The effective charge on CSNPs or nanomag®-D-spio, with or without a pre-formed plasma protein corona, was measured at pH 7.4 in RPMI-1640 without FBS using the Beckman-Coulter DelsaNanoC system.

**Magnetic resonance (MR) relaxometry measurements**

Longitudinal (R_1_) and transverse (R_2_) relaxation rate measurements at 0.47 and 1.41T were obtained on Minispec Mq 20 and Mq 60 spin analyzers (Bruker, Karlsruhe, Germany) and nuclear magnetic relaxation dispersion (NMRD) profiles were recorded on a Spinmaster-FFC 2000 relaxometer (Stelar SRT, Mede, Italy). The measurements were performed on 300 μL of aqueous suspensions of the different nanoparticles with concentrations in the range of 2–7 mM/L Fe.

**Endotoxin assay**

The nanoparticles were controlled for lipopolysaccharide (LPS) contamination prior to biological experiments by using the chromogenic LAL test method (Limulus Amebocyte Lysate endochrome, Charles River Endosafe, Charleston, SC). The LPS levels were always below 50 pg/mL.

**Human plasma**

Buffy coats, citrate – phosphate – dextrose (CPD) treated to prevent blood clotting, were obtained from healthy adult blood donors at the Karolinska University Hospital, Stockholm, Sweden. Using density gradient centrifugation on Lymphoprep (Axis-Shield, Oslo, Norway) the plasma was separated from the cellular component of the buffy coats. Obtained as such, plasma from 14 donors was pooled, aliquoted and stored at −80°C until used. This pooled plasma was used throughout the study. Upon thawing the plasma was centrifuged at 50g, 2 min to remove the presence of any protein precipitates. Plasma was used immediately upon thawing and was never re-frozen.

**Human monocyte-derived macrophages**

Peripheral blood mononuclear cells (PBMCs) were prepared from buffy coats obtained from healthy blood donors (Karolinska University Hospital, Stockholm, Sweden) by density gradient centrifugation using Lymphoprep (Axis-Shield, Oslo, Norway), as described [4]. Thereafter, the PBMCs were positively selected for CD14 expression using CD14 MicroBeads (Miltenyi Biotec, Bergisch Gladbach, Germany). To obtain human monocyte-derived macrophages (HMDMs), CD14^+^ monocytes were cultured for three days in RPMI-1640 medium (Sigma Aldrich) supplemented with 2 mmol/L L-glutamine, 100 IU/mL penicillin, 100 μg/mL streptomycin, and 10% heat-inactivated FBS, all from Gibco Invitrogen Corporation (Paisley, UK), supplemented with 50 ng/mL recombinant macrophage-colony-stimulating factor (M-CSF) (Novakemi, Handen, Sweden). The HMDM phenotype was confirmed by analyzing the F4/80 (Abcam, Cambridge, UK) expression by flow cytometry (FACSCalibur, Becton Dickinson, Franklin Lakes, NJ).

**Cell viability**

Cell viability was assessed by measurement of mitochondrial function using 3-(4,5-dimethyldiazol-2-yl)-2,5 diphenyl-tetrazolium bromide (MTT) (Sigma Aldrich), as described previously [5]. To this end, CD14^+^ monocytes were seeded into 96-well plates as a concentration of 1.0x10^6^ cells/mL and differentiated into HMDM as described above. Cells were then exposed to nanoparticles with or without a protein corona at the indicated concentrations and time-points. No fetal bovine serum (FBS) was added to the cell culture medium when experiments were conducted with particles with or without a plasma protein corona (the viability of HMDMs in the absence of FBS was verified in pilot experiments). MTT conversion was quantified by measuring the absorbance at 570 nm using a spectrophotometer (Infinite F200, Tecan, Männedorf, Switzerland).

**Cytokine secretion**

Cell culture supernatants were harvested at the indicated time-points after exposure of HMDMs to nanoparticles with/without a corona and kept at −80 °C until further analysis. Supernatants from cells incubated with lipopolysaccharide (LPS) (0.1 µg/mL) (Sigma Aldrich) were included as a positive control. TNF-α release was determined by ELISA (Mabtech, Nacka, Sweden) according to the manufacturer's instruction. The absorbance was measured at 405 nm using a spectrophotometer (Infinite F200, Tecan, Männedorf, Switzerland). Results are expressed as pg/mL.

**Cellular internalization of nanoparticles**

**Transmission electron microscopy (TEM).** Cellular uptake of nanoparticles with/without a protein corona was assessed by TEM, as previously described [5]. In brief, HMDMs were seeded at a concentration of 2.0x10^6^/ml and exposed to 50 μg/mL of nanoparticles. No FBS was present in the cell culture medium in order to exclude any effects of FBS proteins. At the indicated times, samples were harvested and fixed and sectioned as previously described. Samples were examined in a Tecnai 12 Spirit Bio TWIN TEM (Fei Company, Eindhoven, The Netherlands) at 100 kV. Digital images were taken using a Veleta camera (Olympus Soft Imaging Solutions, GmbH, Münster, Germany). **Inductively coupled plasma-mass spectrometry (ICP-MS).** For quantification of cellular internalization of nanoparticles, cellular uptake of iron was measured. To this end, HMDM were exposed to 50 μg/mL of nanoparticles with/without a corona for the indicated time-points in serum-free medium. Cell pellets were then harvested and kept at −80 °C until further analysis. Measurement of cellular iron content was performed using ICP-MS (Agilent 7500ce, or Agilent 7700x, Agilent Technologies, Tokyo, Japan) as described previously [6]. The instruments were calibrated using standard reference materials, as described previously [6]. The cellular iron content per 1000 cells is expressed as the percentage of the content in control cells.

**Nanoparticle-protein corona separation**

Nanoparticles were incubated with pooled human plasma under vortexing (20 rotations/min) for 1 h at 37°C. The concentrations of the nanoparticles were 1 mg/ml sample weight (32 µg/ml Fe) and 2 mg/ml sample weight (207 µg/ml Fe) for CSNPs and nanomag®-D-spio, respectively. The concentration of the sample was optimized in order to ensure that the quantity of proteins obtained from the nanoparticles (i.e., the so-called ‘hard’ corona) was detectable using Pierce^®^ BCA Protein Assay (Thermo Scientific). Centrifugation was used to obtain the nanoparticle - protein corona complexes. Due to different specific densities of nanomag®-D-spio (1.4 g/cm^3^) and CSNPs (> 5.26 g/cm^3^) the relative centrifugal force (RCF) used to separate them from the supernatant plasma differed: 3.500g for CSNPs and 70.000 g for nanomag®-D-spio (15 min in each case, at 4°C). The pellets were then re-suspended in 500 µl PBS and centrifuged again for 15 min at the same centrifugal forces. The procedure was repeated three times. After the third washing step the supernatant did not contain any detectable amount of proteins. In order to verify that the centrifugation step itself (the use of different RCF during the separation from plasma and removal of the less tightly bound ‘soft’ protein coronas from the particles) would not exert a selective effect on the composition of the hard corona we introduced two additional controls. Thus, the same starting volumes of pooled plasma as the volume used for incubating the CSNPs and nanomag®-D-spio were included, centrifuged at the same RCF as the particles and washed according to the same washing steps as the corresponding particles; these samples are referred to as ‘plasma CSNP’ and ‘plasma NMAG’. We also included as control two aliquots of the pooled plasma.

**Protein corona visualization**

For the visualization of the protein corona on the surface of the CSNPs, 20 µl of CSNP-protein corona suspension was dropped on a TEM grid and the excess was wicked off with filter paper after 60 s. Negative staining was obtained by adding 20 µl of freshly prepared 0.5% pH 7.0 phosphotungstic acid (PTA) to the sample on the TEM grid (with or without fixation with glutaraldehyde 1% 20 µl, 20 min). The excess of PTA was blotted by subsequent air drying of the grid.

**On-particle digestion and sample preparation**

The nanoparticle-protein corona complexes and controls samples were suspended in a final volume of 190 µl 0.5 M triethylammonium bicarbonate buffer (TEAB) and 9.5 µl 2% sodium dodecyl sulphate (SDS) for 15 min at 90°C. Then, the samples were incubated with 19 µl dithiothreitol (DTT) 100 mM, for 1 h at 60°C. Following incubation with iodoacetamide (IAA) 1 M, for 10 min at RT, the bound proteins were digested with trypsin (MS gold, Promega) (0,1 µg/µl) overnight at 37°C (trypsin:protein ratio 1:50). The trypsinized proteins were recovered after centrifugation of the nanoparticle suspension for 30 min at 3.900 g and 70.000 g, respectively (CSNPs and nanomag®-D-spio). Prior to iTRAQ labeling of the samples an aliquot of each sample was kept for non-labeled analyses. Salts and nonionic detergents were removed from the non-labeled samples using strong cation exchange (SCX) 2-10 µL stage tips (Proxeon) as described below for the SCX microcolumns. Labeling using iTRAQ 8-plex labels were performed according to the manufacturer’s protocol (Applied Biosystems). Pooled iTRAQ labelled digests were applied to 1 ml Strata X-C 33 µm polymeric strong cation exchange (SCX) microcolumns (Phenomenex). The microcolumns were initially washed with 100% methanol followed by MilliQ grade water. The sample was adjusted to >0.1% formic acid and then applied to the columns. After washing with 30% methanol and 0.1% formic acid the samples were eluted with 30% methanol and 5% ammonium hydroxide. Samples were dried in a SpeedVac and submitted to MS. A schematic overview of the protein corona analysis can be found in Fig. 1C.

**Mass spectrometry analysis**

The mass spectrometry analysis was performed essentially as described previously [7]. Briefly, unlabeled samples were dissolved in 10 µL 3% ACN, 0.1% formic acid and iTRAQ sample was dissolved in 100 µL 3% ACN, 0.1% formic acid prior to analysis. Four microliters of labeled sample or two microliters unlabeled were analyzed with a hybrid LTQ-Orbitrap Velos mass spectrometer (Thermo Scientific). An Agilent HPLC 1200 system (Agilent) was used to provide the 70 min gradient (unlabeled samples) and 90 min (iTRAQ samples) gradient. Data-dependent MS/MS (centroid mode) followed in two stages: first, the top-5 ions from the master scan were selected for collision induced dissociation with detection in the ion trap (ITMS); and then, the same 5 ions underwent higher energy collision dissociation (HCD) with detection in the Orbitrap (FTMS). The data were searched by Sequest [8] under the Proteome Discoverer 1.3.0.339 software (Thermo Scientific) against the Swissprot protein sequence database (version 20120202) using a 99% confidence cut-off limit. The proteomics data generated in this study have been deposited to the ProteomeXchange Consortium ([http://proteomecentral.proteomexchange.o​rg](http://proteomecentral.proteomexchange.org)) via the PRIDE partner repository [[9](#_ENREF_27)] with the dataset identifier PXD000766.

**Bioinformatics analysis**

**Statistical analysis.** The number of peptide spectrum-matches (PSMs) for each identified protein was used as quantitative value and were loaded in the R statistical analysis software for further analysis [10]. The PSMs were summed on the Uniprot protein body giving a total of 309 unique proteins (S1 Table). Counts were analyzed using the R statistical programming language version 3.0.2 (2013-09-25) and Bioconductor version 2.15.0 package limma_3.18 and edgeR_3.4.1.[11,12]. For statistical analysis the PSM spectral count values were filtered to only include proteins with data from a minimum of two replicates at a level of at least 0.5 PSMs per thousand, and then formatted as a DGEList object, giving 167 proteins to analyze. The limma function voom [[11](#_ENREF_29)] was used to process count data for analysis using limma/eBayes statistics (loess span parameter was 0.75 for mean-variance trend estimation), robust fitting of the linear model and Benjamini-Hochberg multiple testing corrections (S2 Table). The comparisons included CSNPs versus plasma, nanomag®-D-spio versus plasma, and CSNPs versus nanomag®-D-spio. **Estimation of relative protein abundances.** Relative abundances of proteins in analytes were estimated using the top3 method, which is based on the precursor area of the top 3 most abundant peptides [13], and filtered to include only proteins with relative abundance greater than 0.5% of the total estimated protein amount, giving 90 proteins (S3 Table). **Clustering analysis.** For clustering, Z-score transformed PSM values were used after filtering to only include proteins used in the statistical analysis above and further filtering, based on the top3 calculation as described above, leaving 64 proteins. The PSM spectral counts were log2 transformed, after offsetting by 0.5, converted to Z-scores in a row-wise manner setting the Z-score of PSMs = 0 to zero. Proteins were divided into five similar groups using the R package cluster 1.14.1 and the partitioning around medoids (PAM) method [14] using the “manhattan” distance metric (S4 Table). Clustering was visualized as a heatmap with R, separately hierarchically clustering groups identified by the PAM analysis.^15^ *GO and KEGG enrichment analysis.* KEGG (Kyoto Encyclopedia of Gene and Genomes) Pathway and Gene Ontology (GO) enrichment analysis was performed with the topGO v.1.0 R/Bioconductor package [15] using the” parentChild” method for statistical significance [16], using a threshold of p<0.01 and at least 3 significant proteins (S5-S6 Tables) using protein lists from the PAM clustering analysis (S4 Table) and from the statistical analyses (S2 Table). The total list of all 309 proteins observed in any of the experiments was used as the background for the overrepresentation analysis. Significantly enriched KEGG pathways were detected using the webgestalt tool [17], Fisher’s exact test and a cut off of multiple testing adjusted p-value<0.05 (S7 Table). Significant GO categories and KEGG pathways from the CSNP *versus* plasma comparison were visualized as stacked bar plots with the ggplot2 version 0.9.3.1, using relative abundances of the proteins, separately from each biological replicate (data were derived from S5-S7 Tables, signature used: “Csnp_vs_Plasma_UP_exclude_nMag_UP”). The height of the bar for each protein corresponds to its relative abundance, as determined by the top3 method (S3 Table).

**References**

1. Park J, An K, Hwang Y, Park J-G, Noh H-J, Kim J-Y, et al. Ultra-large-scale syntheses of monodisperse nanocrystals. Nat Mater. 2004;3(12):891-5. doi: 10.1038/nmat1251.
2. Vogt C, Toprak M, Muhammed M, Laurent S, Bridot J-L, Müller R. High quality and tuneable silica shell–magnetic core nanoparticles. Journal of Nanoparticle Research. 2010;12(4):1137-47. doi: 10.1007/s11051-009-9661-7.
3. Stöber W, Fink A. Controlled Growth of Monodisperse Silica Spheres in the Micron Size Range. J Colloid Interface Sci. 1968;26:62--9. doi: 10.1016/0021-9797(68)90272-5.
4. Witasp E, Kupferschmidt N, Bengtsson L, Hultenby K, Smedman C, Paulie S, et al. Efficient internalization of mesoporous silica particles of different sizes by primary human macrophages without impairment of macrophage clearance of apoptotic or antibody-opsonized target cells. Toxicology and Applied Pharmacology. 2009;239(3):306-19. doi: 10.1016/j.taap.2009.06.011.
5. Kunzmann A, Andersson B, Vogt C, Feliu N, Ye F, Gabrielsson S, et al. Efficient internalization of silica-coated iron oxide nanoparticles of different sizes by primary human macrophages and dendritic cells. Toxicology and Applied Pharmacology. 2011;253(2):81-93. doi: 10.1016/j.taap.2011.03.011.
6. Krais A, Wortmann L, Hermanns L, Feliu N, Vahter M, Stucky S, et al. Targeted uptake of folic acid-functionalized iron oxide nanoparticles by ovarian cancer cells in the presence but not in the absence of serum. Nanomedicine. 2014;10(7):1421-31. doi: 10.1016/j.nano.2014.01.006.
7. Pernemalm M, Lehtiö J. A Novel Prefractionation Method Combining Protein and Peptide Isoelectric Focusing in Immobilized pH Gradient Strips. Journal of Proteome Research. 2013;12(2):1014-9. doi: 10.1021/pr300817y.
8. Eng JK, McCormack AL, Yates JR. An approach to correlate tandem mass spectral data of peptides with amino acid sequences in a protein database. J Am Soc Mass Spectrom. 1994;5(11):976-89. doi: 10.1016/1044-0305(94)80016-2.
9. Vizcaíno JA, Côté RG, Csordas A, Dianes JA, Fabregat A, Foster JM, et al. The Proteomics Identifications (PRIDE) database and associated tools: status in 2013. Nucleic Acids Research. 2013;41(D1):D1063-D9. doi: 10.1093/nar/gks1262.
10. Team RDC (2011) R: A language and environment for statistical computing. R Foundation for Statistical Computing, Vienna, Austria**,** URL: www.R-project.org ISBN 3-900051-07-0.
11. Law CW, Chen Y, Shi W, Smyth GK. Voom: precision weights unlock linear model analysis tools for RNA-seq read counts. Genome Biol. 2014;15(2). doi: 10.1186/gb-2014-15-2-r29.
12. McCarthy DJ, Chen Y, Smyth GK. Differential expression analysis of multifactor RNA-Seq experiments with respect to biological variation. Nucleic Acids Res. 2012;40(10):4288-97. doi: 10.1093/nar/gks042.
13. Silva JC, Gorenstein MV, Li G-Z, Vissers JPC, Geromanos SJ. Absolute quantification of proteins by LCMSE: a virtue of parallel MS acquisition. Mol Cell Proteomics. 2006;5(1):144-56. doi: 10.1074/mcp.M500230-MCP200.
14. Kaufman L, Rousseeuw PJ (1990) Finding Groups in Data: An Introduction to Cluster Analysis. JohnWiley & Sons: New York, USA.
15. Gentleman RC, Carey VJ, Bates DM. Bioconductor: Open software development for computational biology and bioinformatics. Genome Biol. 2004;5:R80. doi: 10.1186/gb-2004-5-10-r80.
16. Grossmann S, Bauer S, Robinson PN, Vingron M. Improved detection of overrepresentation of Gene-Ontology annotations with parent–child analysis. Bioinformatics. 2007;23(22):3024-31. doi: 10.1093/bioinformatics/btm440.

17. Duncan DT, Prodduturi N, Zhang B. WebGestalt2: an updated and expanded version of the Web-based Gene Set Analysis Toolkit. BMC Bioinformatics. 2010;11(Suppl 4):P10. doi: doi:10.1186/1471-2105-11-S4-P10.
